# Role of First-Row *d*‑Block Metals in Shaping the Properties of Nonstoichiometric SrTiO_3_

**DOI:** 10.1021/acs.inorgchem.6c02457

**Published:** 2026-07-21

**Authors:** Agnieszka Łącz, Beata Bochentyn, Tadeusz Miruszewski, Ewa Drożdż

**Affiliations:** † Department of Inorganic Chemistry, Faculty of Materials Science and Ceramics, AGH University of Krakow, al. A. Mickiewicza 30, Krakow 30-059, Poland; ‡ Institute of Nanotechnology and Materials Science, Faculty of Applied Physics and Mathematics, Advanced Materials Centre, 49557 Gdańsk University of Technology, Gabriela Narutowicza 11/12, Gdańsk 80-233, Poland

## Abstract

The A-site nonstoichiometry in doped ABO_3_ perovskites is typically introduced to tailor the materials’ functional properties through defect engineering and to promote dopant exsolution. In this work, the redox behavior and electrical properties of 5 mol % Sr-deficient strontium titanate doped with the first-row *d*-block elements (Mn, Fe, Co, Ni) were investigated to elucidate the relationships between dopant characteristics and the resulting properties of the final materials. The materials were sintered at 1200 °C and 1400 °C to obtain samples with significantly different microstructures. The results indicate that although the B–O bond energy contributes to the overall physicochemical behavior of the investigated materials, the properties of SrTiO_3_-based systems are generally shaped by the nature of the dopant and, more specifically, by its intrinsic characteristics. In Fe- and Ni-doped materials, the key factor is the ability of these cations to be exsolved as metallic particles under reducing conditions. In Co-containing materials, the physicochemical behavior is strongly affected by the volatility of cobalt species at high temperatures. In contrast, Mn-doped systems are mainly influenced by temperature-dependent site preferences of Mn ions, which can occupy both the Sr and Ti sublattices.

## Graphical abstract

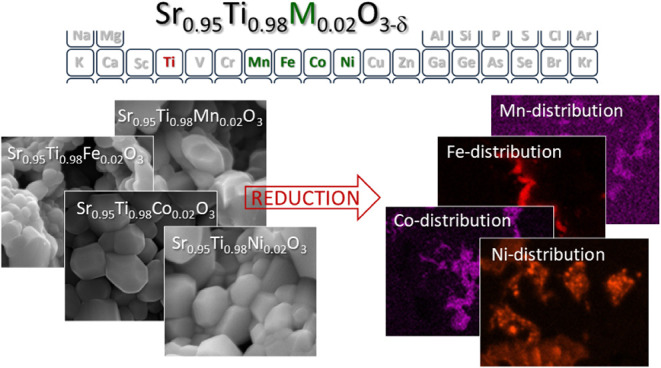

## Introduction

Recently, increasing attention has been paid to the Sr-site-deficient SrTiO_3_.^1–5^ This interest is strongly related to exsolution phenomena,^6,7^ since a deficiency of A-sublattices in the ABO_3_ perovskite structure is assumed to promote the exsolution of transition-metal dopants initially incorporated into the B-sublattice, leading to the formation of active metal nanoparticles on the perovskite surface.^8–10^ Also, the A-site deficiency in the ABO_3_ perovskites is known to increase the concentration and mobility of oxygen vacancies in the lattice oxygen.^11,12^

The defect structure of SrTiO_3_ is strongly dependent on the temperature and the oxygen partial pressure (pO_2_) of the surrounding atmosphere. Changes in defect chemistry induced by changes in pO_2_ can be systematically analyzed using Brouwer diagrams.^13–15^ Generally, in undoped SrTiO_3_, at high pO_2_, electron holes are the dominant charge carriers. In contrast, under low pO_2_ conditions, the formation of oxygen vacancies and electron generation, as a result of charge compensation, is favored. The thermodynamic calculation indicates that at temperatures above 900 °C, oxygen vacancies are the dominant charge carriers in undoped SrTiO_3_ over a wide pO_2_ range (10^–11^–10^–1^ bar).^13^ However, a further increase in temperature also enhances the concentration of additional defects: strontium vacancies and electron holes (high pO_2_) and electrons (low pO_2_). Although in stoichiometric SrTiO_3_, oxygen vacancies are reported as the dominant native defects,^15,16^ its electrical conductivity remains very low. In nonstoichiometric Sr_1–*x*_TiO_3_, the main defects are strontium and oxygen vacancies,^4,17^ which are expected to enhance electrical conductivity and increase the contribution of oxygen-ion transport relative to stoichiometric SrTiO_3_. However, the defect structure of Sr-deficient strontium titanate appears more complex, as additional lattice charge compensation may lead to local structural rearrangements, including partial substitution of Ti for Sr-sites, which generate donor states due to differences in oxidation states.^18,19^

A second route to modifying the defect structure of perovskite materials is aliovalent doping. In SrTiO_3_, it can be realized by introducing dopants into both the Sr and Ti sublattices. Concerning the material’s reducibility, the doping at the titanium sublattice seems to be more interesting since metals of the first-row of *d*-block (Mn, Fe, Co, Ni)—which can relatively easily change their oxidation state and often can take various oxidation states—are considered as the proper dopant into the Ti-site mainly due to the geometric factor (ionic radii). When incorporated into the strontium titanate structure, these cations typically act as acceptor-type dopants,^20–27^ primarily generating electron holes and promoting the formation of oxygen vacancies. However, special attention should be paid to manganese, as it can be incorporated into both Ti- and Sr-sites.^28–30^ Manganese typically adopts + II and + IV oxidation states when incorporated into the strontium and titanium sublattices, respectively.^31–33^ Consequently, manganese can act as an isovalent dopant. Its incorporation does not significantly change the SrTiO_3_ defects’ structure, also due to a self-compensation mechanism, which is also observed when manganese occupies Sr- and Ti-sites in the + III oxidation state.^34^ It should be noted that the mechanism of manganese incorporation into the SrTiO_3_ structure depends strongly on the synthesis atmosphere: under oxygen-rich conditions, manganese preferentially occupies Ti-sites, whereas at lower oxygen partial pressure, manganese ions are reduced and subsequently relocate to Sr-sites.^35,36^ Iron incorporated into the titanium sublattice can take mixed + III and + IV oxidation states,^37,38^ leading to oxygen nonstoichiometry.^23^ Cobalt also exhibits multiple oxidation states, primarily + II and + III, when incorporated into the SrTiO_3_ structure,^24,39,40^ although Co^4+^ is also postulated.^41^ The oxidation state of incorporated cobalt strongly depends on its concentration: at low doping levels, cobalt stabilizes the + II oxidation state; as the Co content increases, a higher fraction of cobalt adopts the + III oxidation state.^39^ Although cobalt usually substitutes for the Ti-site in the SrTiO_3_ structure, its position is reported to depend strongly on the synthesis temperature: at high temperatures (1600 °C), Co^2+^ is dominant and occupies Sr-sites.^25^ The presence of nickel in multiple oxidation states in perovskite structures has also been documented.^42–44^ When incorporated into SrTiO_3_, nickel usually takes the + II oxidation state,^26,45,46^ although occurrences of nickel in the + III^47^ and + IV^27^ oxidation states have also been reported. Furthermore, the synthesis method may influence the nickel oxidation state, as high concentrations of organic precursors or additives can create reducing conditions that promote the incorporation of nickel in the + II rather than + III oxidation state.^26^ It should also be added that the defects’ structure of doped SrTiO_3_ is a complex topic since the associates’ defects, such as Me_Ti_″–V_O_^••^, are also postulated for materials doped with Mn and Co.^25,48^

The maximum solubility of first-row *d*-block metals in the SrTiO_3_ structure depends on several factors, among which the synthesis method, synthesis temperature, and atmosphere play dominant roles. According to the literature, Mn solubility in the Ti-sublattice can exceed 15 mol %^49^, whereas its solubility in the Sr-sublattice is significantly lower, typically reaching 2–3 mol %.^49,50^ In contrast, the incorporation of iron into the SrTiO_3_ structure can occur over a wide range, as solid-solution formation across the entire range from SrTiO_3_ to SrFeO_3_ has been reported in the literature.^23,37^ Similarly, the relatively high solubility of cobalt in the SrTiO_3_ structure has been documented, reaching up to 40 mol %^38^ or even 90 mol %^51^ in the titanium sublattice, which is a result of similarities between the SrTiO_3_ and SrCoO_3_ structures. Nevertheless, for materials intended for electrochemical applications, substantially lower Co/Fe content (typically below 8–10 mol %) is commonly applied to maintain structural stability and functional performance.^25,39,52^ The solubility limit of nickel in the strontium titanate structure is generally reported to be between 2 and 4 mol %.^42,43^ However, in a multidoped SrTiO_3_-based system with Sr deficiency, Ni incorporation can be increased to 10 mol %.^45^

The literature contains numerous studies on the effective incorporation of dopants into the strontium titanate structure, primarily to improve its electrical properties. Such studies typically investigate a series of materials with varying dopant concentrations or combinations of multiple dopants. In contrast, the present work focuses on comparing the properties of SrTiO_3_-based materials doped with first-row *d*-block elements (Mn, Fe, Co, Ni) and elucidating potential correlations between their electrical and reduction properties. Moreover, the basic chemical properties of the dopants, including electronegativity, reduction potential, and metal–oxygen bond energy, are also analyzed.

## Materials and Methods

### Sample Preparation

As recommended in the literature, all materials were synthesized with a controlled Sr-sublattice deficiency (5 mol %) to support the incorporation of dopants into the perovskite structure. To assess and compare the influence of each dopant across the entire material series, a constant doping level was required, regardless of the element introduced. Therefore, a uniform dopant concentration of 2 mol % (relative to the Ti content) was applied for all compositions. Concerning both the controlled Sr deficiency and the low dopant concentration, it can be assumed that, regardless of the element introduced (Mn, Fe, Co, or Ni), the effective doping level would be the same throughout the entire series.

Materials with the general formula Sr_0.95_Ti_0.98_M_0.02_O_3_, where M = Mn, Fe, Co, and Ni, were synthesized using the citrate method to obtain powders, and the appropriate treatment was applied to obtain sintered materials. It should be noted that the formula Sr_0.95_Ti_0.98_M_0.02_O_3_ (used to label the materials) reflects only the nominal composition of the materials; the oxygen sublattice deficiency induced by both Sr deficiency and *d*metal doping, although possible, is not included in this general formula.

The detailed scheme of the procedure is presented in Figure 1. The chemical reagents were supplied by Acros Organics (Sr­(NO_3_)_2_, Fe­(NO_3_)_3_·6H_2_O, Co­(NO_3_)_2_·6H_2_O, Ni­(NO_3_)_2_·6H_2_O, titanium­(IV) isopropoxide), POCh (methanol, citric acid monohydrate), or ChemPur (Mn­(CH_3_COO)_2_·4H_2_O) and had an analytical grade except of titanium­(IV) isopropoxide, whose chemical purity was 98.0%. Based on the solid metal nitrates (anhydrous or hydrated), aqueous solutions of Sr­(NO_3_)_2_, Fe­(NO_3_)_3_, Co­(NO_3_)_2_, and Ni­(NO_3_)_2_ at approximately 1 M were prepared. The exact concentrations of the solutions were determined by classical gravimetric analysis. As the manganese precursor, manganese acetate tetrahydrate in the solid state was used. In the synthesis, the methanol was used instead of water to avoid titanium hydrolysis. Citric acid was used as the complexing agent for metal ions; the molar ratio of citric acid to metal cations was 1.5:1. All the reagents were subsequently added to the methanol-citric acid mixture, heated under constant stirring on a magnetic stirrer to obtain a clear solution, and the solvents were then evaporated. It was followed by the densification of the precursor mixture (250 °C/12h). The received powders were crushed in an agate mortar and calcined in air at 900 °C (1 °C min^–1^) for 3 h. The obtained powder was homogenized in agate mortars and formed into pellets (φ = 10 mm) by uniaxial pressing (5 MPa). Two series of materials differing in sintering temperature were finally prepared: series 1 was sintered at 1200 °C, and series 2 at 1400 °C. The other sintering conditions were the same: air atmosphere; heating rate of 5 °C min^–1^ from RT to 800 and 3 °C min^–1^ from 800 to 1200 °C/1400 °C; sintering time of 3 h.

**Figure 1:**
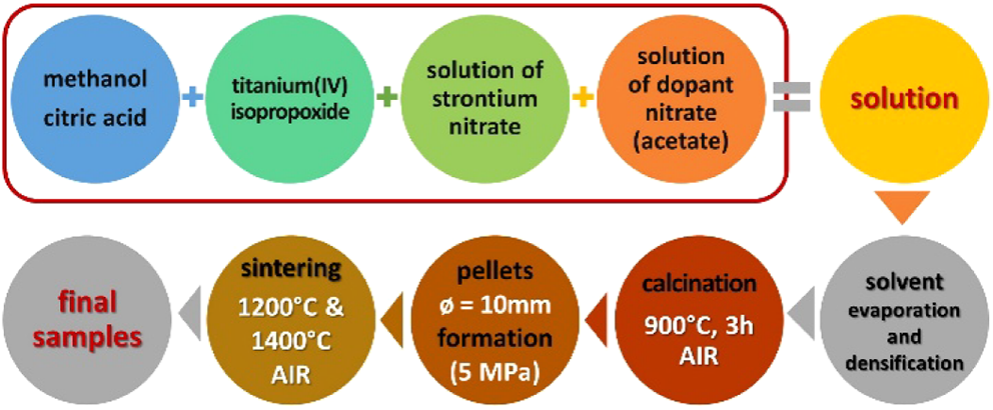
Diagram illustrating the synthesis procedure for Sr_0.95_Ti_0.98_M_0.02_O_3_ (M = Mn, Fe, Co, and Ni) material. A red rectangle highlights the composition of the initial precursors’ solution, while arrows indicate the subsequent synthesis steps.

### Dopant Characterization

Four dopants, Mn, Fe, Co, and Ni, were used as modifiers for nonstoichiometric strontium titanate. Their basic properties are presented in Figure 2. One can notice that the number of valence electrons, electronegativity, and redox potential change as we gradually move from Mn to Ni. Additionally, all of these modifiers can adopt various oxidation states (typically + II, + III, or + IV) when incorporated into the Ti-site in the strontium titanate structure. Thus, this factor can also influence the final material properties. The Supporting Information (Table S1) lists the ionic radii of the dopants for each oxidation state.^53^ It should also be noted that changes in dopant oxidation state can occur easily, especially for Mn, Co, and Fe. Therefore, additional redox reactions (not only M^2+^ + 2e′ = M^0^ as shown in Figure 2) should be considered when the redox/electrical properties of the synthesized materials are discussed. It should be emphasized that the given redox potential refers to an aqueous solution and thus does not necessarily illustrate the behavior of cations in solid-state oxides. In perovskite systems, the redox activity of the B-site cations is more commonly discussed in terms of oxygen-vacancy formation energy. Unfortunately, this parameter is not constant; it depends on many factors, including the A-site composition, oxygen partial pressure, and temperature. Also, the B–O bond energy in BO_6_ octahedra, which is closely related to the oxygen-vacancy formation energy, may also be considered as an indicator of the cation’s reducibility. Nevertheless, these energies are also influenced by many factors, thus it is hard to provide exact values from the literature. However, an attempt to predict the chemical stability of the perovskite based on the average metal–oxygen bond energy ABE has already been reported in the literature.^54,55^ In this model, the stability of the structure is primarily controlled by the bond energy between the B-site metal and oxygen, with a secondary contribution from the A-site metal–oxygen bond energy. Based on this model, the B–O bond energy (related to the BO_6_ octahedra) can be calculated. Additionally, the stability of the perovskite structure is strongly correlated with the Goldschmidt tolerance factor, *t*.^56,57^ The B–O bond energy and *t* values, calculated assuming that the dopant (Mn, Co, Fe, and Ni) adopts a + II oxidation state in the Sr-deficient SrTiO_3_ structure, are shown in Figure 2, and those for other oxidation states are given in Table S2 in the Supporting Information. The equation used for B–O bond energy and *t* calculations, along with methodological details, is provided in Supporting Information.

**Figure 2:**
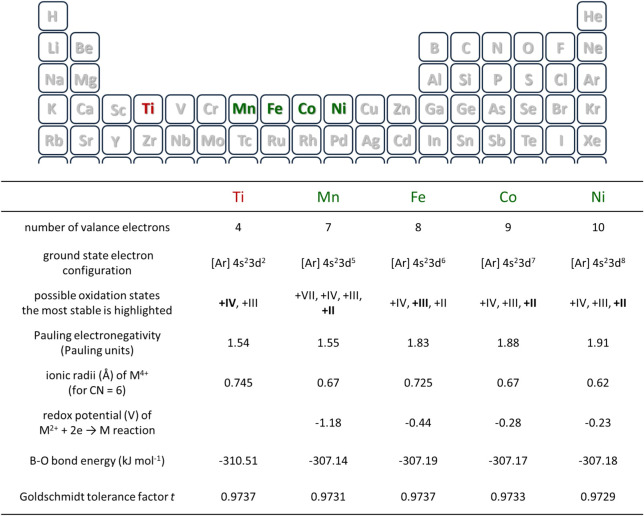
Comparison of selected electronic and physicochemical properties of Ti and *d*-metal dopants (Mn, Fe, Co, and Ni), including the number of valence electrons, ground-state electron configuration, possible oxidation states, Pauling electronegativity, ionic radii of M^4+^ ions (for CN = 6), redox potentials (M^2+^/M), B–O bond energies, and Goldschmidt tolerance factors.

## Methods

X-ray diffraction (XRD) measurements were performed to analyze the phase composition of the synthesized materials and structural parameters of the observed phases. The diffractograms were recorded on a Philips X’Pert Pro diffractometer (Cu–K_α_ radiation, 1.5406 Å) at a rate of 0.012 deg s^–1^ over the 2θ range from 10 to 90 °. For quantitative analysis of phase composition and determination of lattice parameters, Rietveld refinement was performed using the HighScore Plus software.

Microstructural analysis was performed using SEM micrographs recorded on a Scios 2 scanning electron microscope (Thermo Fisher Scientific). The observations were performed under high vacuum conditions. The grain size analysis was performed based on the received micrographs. At least 50 grains were analyzed for each material to construct the grain-size distribution. The results were presented as histograms. Additionally, the chemical composition of the materials was examined using energy-dispersive X-ray spectroscopy (EDX). Furthermore, for microstructural characterization, the relative density of the sintered materials was calculated from the samples’ mass, geometry, and the theoretical density of tausonite, 5.14 g·cm^–3^ (according to ICCD no. 98–005–6717). The obtained values were used to determine the porosity.

Specific surface area (SSA) values were determined from low-temperature nitrogen adsorption isotherms obtained using a Micromeritics ASAP 2010 analyzer. Before analysis, the samples were degassed under vacuum (10^–3^ mmHg) for 24 h to remove physically adsorbed species and moisture. The SSA values were calculated using the Brunauer–Emmett–Teller (BET) equation with the five-point approach over the relative pressure (p/p_0_) range of 0.06 to 0.20.

To assess the potential participation of the synthesized materials in reduction and oxidation reactions, temperature-programmed reduction (TPR) and temperature-programmed oxidation (TPOx) measurements were performed using the ChemiSorb 2750 apparatus supplied by Micromeritics. Samples with a mass of about 0.4 g were placed in the quartz reactor and heated at a rate of 10 °C min^–1^ to 900 °C in a flow of 5% H_2_/Ar or 5% O_2_/Ar for TPR and TPOx measurements, respectively. The measurements were performed according to the following procedure: first reduction–oxidationsecond reduction, with a helium flush in between. The detailed route of TPR/TPOx is presented in Supporting Information (Figure S1). The materials after the second reduction were submitted for XRD analysis and SEM observation as part of the postmortem analysis.

The total electrical conductivity of the investigated materials (the sum of electronic and ionic components) was determined as a function of temperature using the DC four-point probe technique (DC-4W), which minimizes the influence of contact resistance and lead impedance. Bar-shaped samples were contacted with four platinum electrodes arranged in a line along the sample length. A constant current was supplied using a Keithley 2401 SourceMeter, while the voltage drop between the inner probes was recorded with a Keysight 34970A data acquisition system equipped with a high-impedance digital voltmeter module. The electrical conductivity was calculated from the measured current and voltage, accounting for the geometric dimensions of each specimen. Temperature-dependent measurements were carried out in a tubular furnace under controlled gas atmospheres. In the former measurement sequence, each sample was heated in dry synthetic air (pO_2_ ≈ 0.2 atm.) from room temperature up to 800 °C at a constant heating rate of 5 °C min^–1^. After reaching 800 °C, the temperature was held for 2 h to ensure thermal equilibration and stabilization of defect chemistry. Subsequently, the sample was cooled stepwise, with isothermal holds at 50 °C intervals. At each temperature step during cooling, the sample was held for 1 h before data evaluation to ensure thermal and electrical stabilization. Electrical data were recorded continuously throughout the entire cooling cycles. After completion of the measurements in air, the same sample was subjected to an identical thermal protocol under a dry hydrogen atmosphere (pO_2_ ≈ 10^–23^ atm). The heating and cooling rates, as well as the isothermal stabilization steps during cooling, were kept unchanged to ensure comparability of the data. The only modification concerned the high-temperature dwell at 800 °C, which was extended to 4 h in hydrogen to allow sufficient equilibration under strongly reducing conditions. The activation energy for the conduction process was extracted from Arrhenius plots of total electrical conductivity versus the inverse temperature, assuming thermally activated transport behavior. It must be underlined that the activation energy for the conduction process was not calculated from all experimental data points but only from selected ones. The selection was performed after ensuring that the sample was electrically and thermally stabilized at each temperature. A detailed illustration of the data point selection procedure is provided in the Supporting Information (Figure S2).

## Results and Discussion

### Microstructure and Structure

Figure 3 presents SEM micrographs and the corresponding average chemical compositions of SrTiO_3_-based materials modified with *d*-block metals, sintered at 1200 and 1400 °C. Additionally, histograms of the grain-size distributions of the sintered materials, along with their porosities, are included in this figure. Materials sintered at 1200 °C exhibit a relatively high porosity (40–50%), irrespective of the dopant used. However, the dopant’s influence is evident in the grain size of the sintered materials. The Mn-doped material has significantly small grains with sizes not exceeding 0.5 μm. Grains of Fe- and Co-doped materials exhibit similar grain-size distributions and ranges, with grains of comparable dimensions. In contrast, the Ni-doped material shows slightly larger grains, up to 2 μm, and a broader grain-size distribution. Increasing the sintering temperature to 1400 °C resulted in predictable microstructural changes—a reduction in porosity and an increase in grain size. For the materials doped with Fe, Co, and Ni, these changes are comparable, with grain sizes increasing by approximately 1.5–2 times and porosity decreasing by about 20–40%. However, it must be noted that for Fe-modified materials, the change in microstructure with temperature is the smallest. In contrast, for the Mn-doped material, the changes in the microstructure were much more significant, with grains reaching up to 2 μm (approximately a 4-fold increase) and porosity decreasing to 9.5%. Such a large change in microstructure, particularly when compared with that observed in the Fe-, Co-, and Ni-doped materials, suggests that the effect may not be only due to the higher sintering temperature but could also indicate structural changes specific to manganese doping.

**Figure 3:**
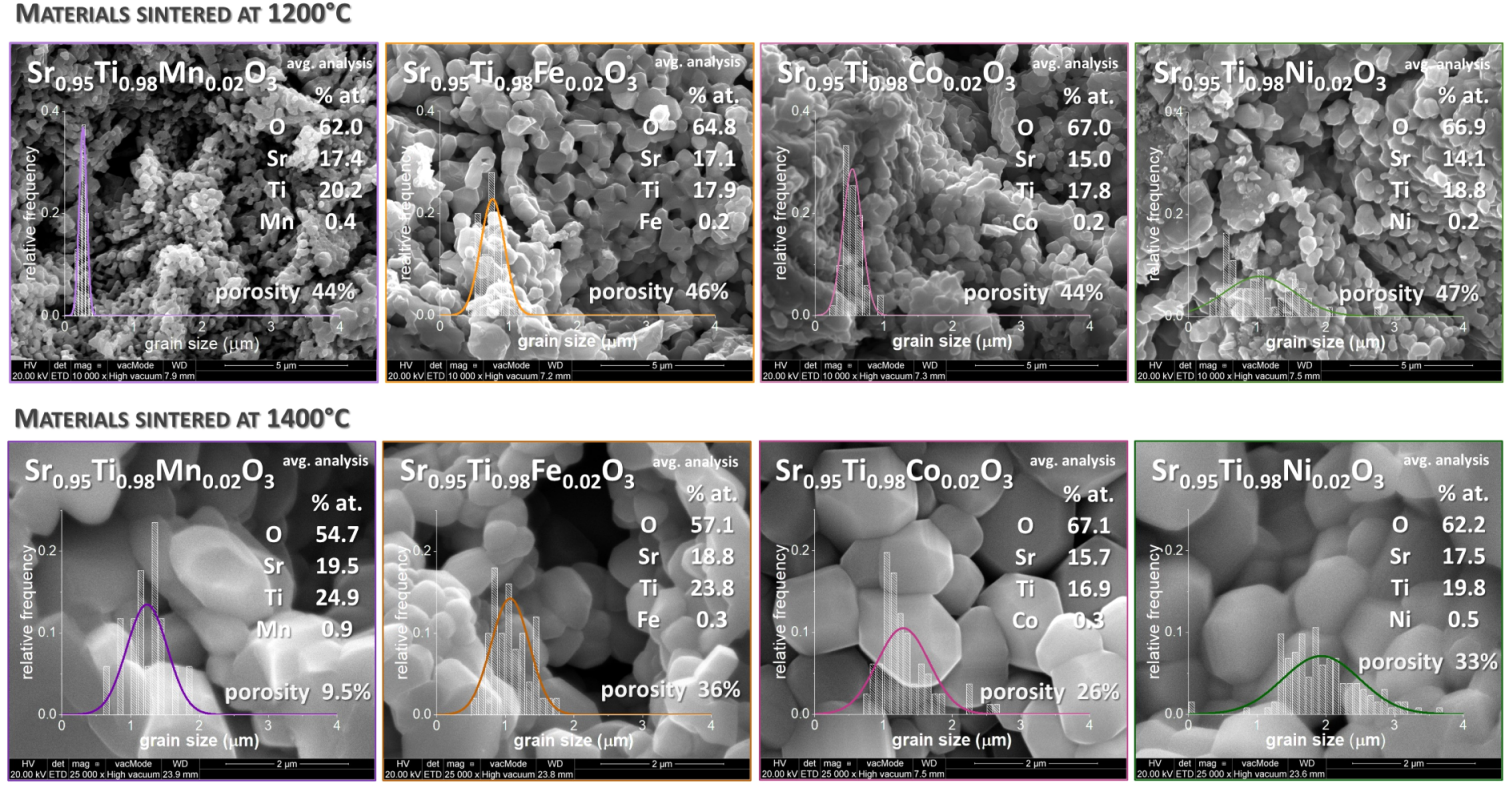
SEM microphotographs of modified SrTiO_3_-based materials sintered at 1200 and 1400 °C, together with the corresponding average chemical composition determined by EDS and the calculated porosity. Histograms of grain-size distributions with fitted distribution curves are included. The grain size was evaluated from the SEM microphotographs.

The materials sintered at 1400 °C have much larger grains, which are due to higher thermal energy during sintering and faster diffusion of O and B-site atoms. Interestingly, at 1400 °C, the sample containing Mn and Co is better sintered and has significantly lower porosity compared to those containing Fe and Ni. To summarize, the significant impact of the used dopant on sintering kinetics and diffusion in SrTiO_3_ was observed.

It should be added that the SSA of materials sintered at 1200 °C was in the range 1.0–1.4 m^2^·g^–1^ for Fe-, Co-, and Ni-modified materials, and reached 2.7 m^2^·g^–1^ for Mn-modified material, while for materials sintered at 1400 °C, it was even lower, below 0.3 m^2^·g^–1^ for all materials. The significantly low SSA values indicate that the porosity clearly seen in Figure 3 is predominantly closed.

The XRD results for materials sintered at 1200 and 1400 °C are shown in Figures 4 and S3 (Supporting Information), where the Rietveld refinement of the X-ray diffraction data is presented, showing the experimental patterns, calculated profiles, and difference curve. All samples contain the cubic phase of strontium titanate (*Pm*-3m; ICSD reference code: 98–005–6717). In addition, the minor secondary phase—tetragonal TiO_2_ (rutile, P42/mnm, ICSD reference code: 98–016–5925) was detected for Fe-, Co- and Ni-doped materials sintered at 1200 °C (1.3%, 1.9% and 1.7% of TiO_2_, respectively) and Sr_0.95_Ti_0.98_Ni_0.02_O_3_ sintered at 1400 °C (0.3% of TiO_2_). The detection of TiO_2_ as an additional phase is not unexpected, as the nominal composition was intentionally set to create a 5% nonstoichiometry in the strontium sublattice. Small amounts of TiO_2_ may be present in other samples but remain undetected because they are below the XRD detection threshold or exist in an amorphous form. Moreover, for Sr_0.95_Ti_0.98_Fe_0.02_O_3_ sintered at 1200 °C, additional diffraction peaks were detected at 30.5°, 44.5°, 50.1°, and 50.7°. Similarly, for Mn-modified material sintered at 1400 °C, very small additional peaks at 26.2°, 29.3°, 31.0°, and 31.4° were also observed. These reflection peaks could not be indexed to any known phases in the Inorganic Crystal Structure Database (ICSD), suggesting the possible formation of unidentified secondary phases or structurally complex phases in the Sr–Ti–Fe–O/Sr–Ti–Mn–O systems. Interestingly, these peaks were not observed in the diffractograms of samples sintered at other temperatures (1400 °C for Fe-doped and 1200 °C for Mn-doped materials), indicating that the structural transformation may occur as sintering temperature increases. This effect is particularly seen in the iron-doped sample, where an increased processing temperature increased the solubility of Fe–Ti–(Sr)–O-based phases in the perovskite structure, resulting in the formation of a single-phase material.

Generally, the incorporation of a dopant into the strontium titanate structure should result in a change in the SrTiO_3_ lattice parameter. However, it should be noted that the formation of the unidentified phases can lead to a lower-than-assumed amount of incorporated dopant. The strontium titanate lattice parameters for unmodified SrTiO_3_ sintered at 1200 and 1400 °C are 3.9070 ± 0.002 Å and 3.9043 ± 0.003 Å, respectively. For the modified SrTiO_3_-based materials, the proper parameters are collected in Table 1.

**Figure 4:**
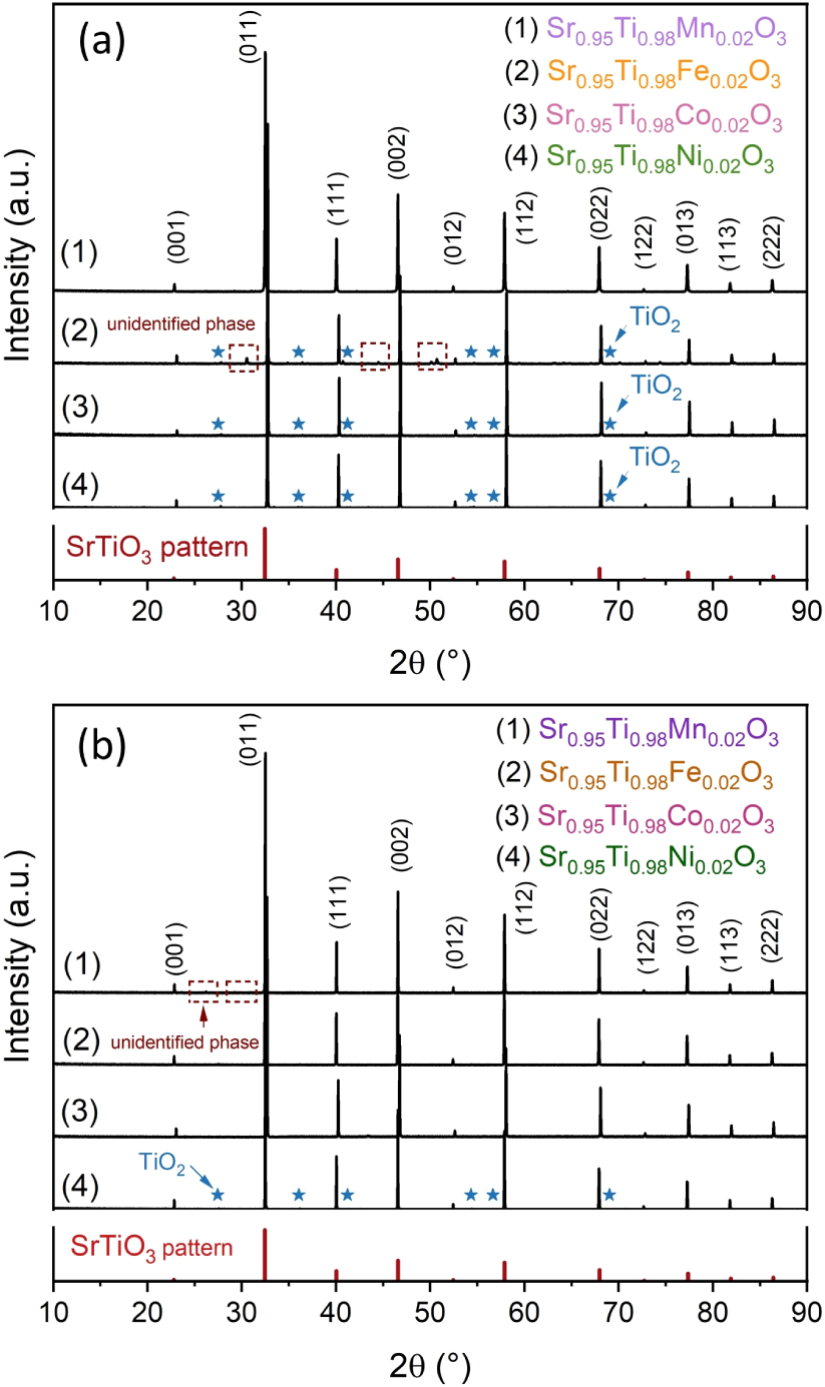
XRD results for modified SrTiO_3_-based materials sintered at 1200 °C (a) and 1400 °C (b), are shown together with the reference pattern of cubic SrTiO_3_ (*Pm*-3m, ICSD reference code: 98–005–6717). The blue stars mark the reflections of a minor TiO_2_ phase (P42/mnm, ICSD reference code: 98–016–5925), while the brown rectangles highlight the unidentified phases.

**Table 1:** Lattice Parameters of Strontium Titanate Obtained from Rietveld Refinement of XRD Data for All Synthesized Materials

	SrTiO_3_ lattice parameter (Å)			
Sintering temp.	Mn-doped	Fe-doped	Co-doped	Ni-doped
1200 °C	3.9037 ± 0.0001	3.9081 ± 0.0002	3.9071 ± 0.0004	3.9068 ± 0.0003
1400 °C	3.9042 ± 0.0003	3.9044 ± 0.0003	3.9062 ± 0.0003	3.9049 ± 0.0002

It should be noted that only a minimal change in the lattice parameters is expected, since the low dopant concentration (2 mol %) is insufficient to induce significant structural changes. The interpretation of these results is also complicated by the fact that these dopants can be incorporated into the perovskite structure in multiple oxidation states. Thus, drawing a clear conclusion about the incorporation of Mn/Co/Fe/Ni and their oxidation states within the perovskite structure is challenging. In particular, the observed change in the lattice parameters relative to undoped SrTiO_3_ falls within the experimental error margin. A relatively significant change in the lattice parameter of SrTiO_3_ is observed only in Mn-doped strontium titanate sintered at 1200 °C and Co-modified strontium titanate sintered at 1400 °C. The decrease in the lattice parameter for Sr_0.95_Ti_0.98_Mn_0.02_O_3_ suggests that manganese, after sintering at 1200 °C, is incorporated into the perovskite structure at the titanium sublattice in a + III or + IV oxidation state, as the ionic radii of Mn^3+^ and Mn^4+^ are smaller than those of Ti^4+^ (Table S1). However, it should be noted that, for geometric reasons, only the low-spin configuration of Mn^3+^ should be considered. An additional factor should also be taken into account when analyzing Mn doping in strontium titanate, especially when nonstoichiometry is introduced into the strontium sublattice. According to the literature,^30,49^ manganese can be incorporated not only into the titanium sublattice but also into the strontium sublattice, predominantly in the + II oxidation state. This incorporation is associated with a decrease in the lattice parameter of strontium titanate. The incorporation of Mn into the Sr or Ti sublattice, depending on the Sr/Ti ratio, was also postulated in our previous studies.^58^ The substitution of strontium by manganese may also be a reason for the observed differences in the microstructure of Mn-modified materials sintered at 1200 and 1400 °C, compared to those doped with Fe, Co, or Ni. It cannot be excluded that the formation of additional phases observed in materials sintered at 1400 °C may also result from the partial migration of Mn cations between the Ti and Sr sublattices. Additionally, the increase in the lattice parameter of Sr_0.95_Ti_0.98_Co_0.02_O_3_ sintered at 1400 °C compared to undoped SrTiO_3_ implies that cobalt, while incorporated into the titanium sublattice, takes a + II oxidation state. A comparison of the relevant ionic radii (Table S1) indicates that Co^2+^ likely adopts a high-spin configuration within the SrTiO_3_ structure.

### Redox Properties

To investigate the redox properties of the synthesized materials and oxidation states of the dopants, temperature-programmed reduction (TPR) measurements were performed. The TPR profiles for the first (TPR I) and second (TPR II) reduction cycles are shown in Figure 5. It is known that the reduction of undoped SrTiO_3_ occurs at temperatures above 600 °C; however, the degree of this process is relatively small and can therefore be neglected in the analysis of TPR profiles recorded for *d*-block metal-doped SrTiO_3_ materials.

**Figure 5:**
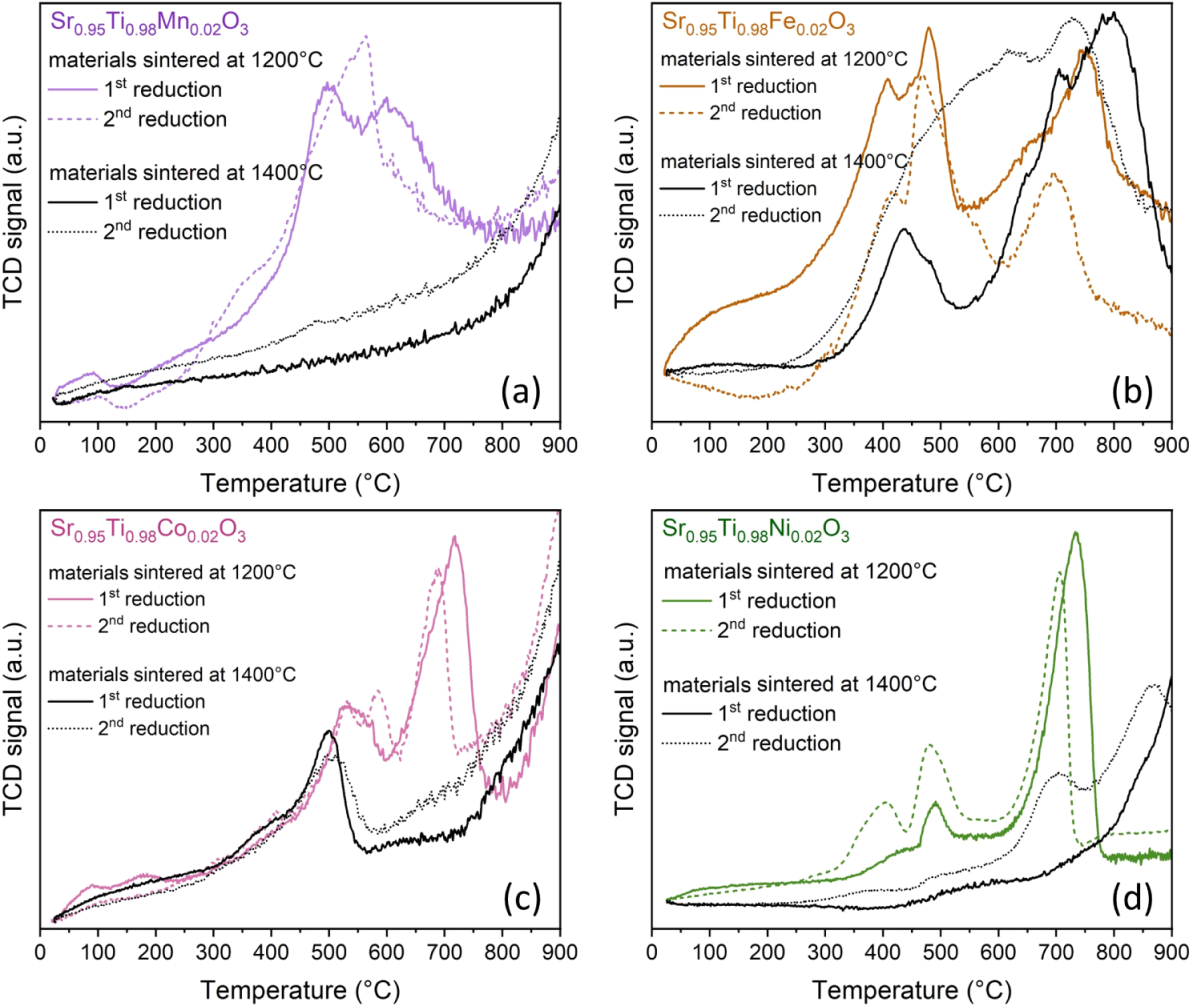
TPR profiles of SrTiO_3_-based materials doped with Mn (a), Fe (b), Co (c), and Ni (d) sintered at 1200 and 1400 °C. The solid lines represent the TPR curve for the first reduction, while the dotted lines correspond to the second reduction. The colored curves denote materials sintered at 1200 °C, whereas the black curves correspond to materials sintered at 1400 °C.

The TPR profiles for manganese-modified materials sintered at 1200 °C (Figure 5a) clearly indicate the occurrence of reduction processes within the materials. It should be noted that the reduction of Mn^2+^ ions occurs at temperatures exceeding 900 °C;^59^ thus, the effects associated with this process cannot be observed in the recorded TPR curves. Nevertheless, the profiles for the materials sintered at 1200 °C show a reduction in manganese ions—Mn^3+^ and Mn^4+^, whose presence in the strontium titanate structure was postulated based on diffraction data. The observed reduction is not fully reversible, as indicated by differences in both the shapes and intensity of the TPR I and TPR II profiles. It should also be added that the + III oxidation state is not stable for manganese. The disproportionation reaction 2Mn^3+^ → Mn^2+^ + Mn^4+^ can easily proceed in this system.^60,61^ Thus, the difference in the shapes of TPR I and TPR II may also result from a change in the Mn^4+^/Mn^3+^/Mn^2+^ ratio in the system after the reduction/oxidation/reduction process. In contrast, the TPR profiles for materials sintered at 1400 °C suggest that manganese in these materials exists predominantly in the + II oxidation state, as no reduction peaks are observed in the temperature range where Mn^3+^/Mn^4+^ reduction occurs. Moreover, the unusual effect is observed in both TPR I and TPR II profiles: a slow, gradual increase in hydrogen consumption. This behavior can also be attributed to oxygen vacancies formed in the materials during high-temperature sintering, arising from the natural tendency of oxygen to leave the oxygen sublattice at elevated temperatures. In general, this effect can also be observed when the samples with low porosity are reduced, as hydrogen diffusion in the material is limited.

The TPR profiles recorded for Fe-doped SrTiO_3_ indicate the complexity and multiplicity of the reduction processes, which occur in two different temperature regions: below 600 °C and above 600 °C (Figure 5b). The lower-temperature region is typically associated with the reduction of iron oxides,^62^ while the higher-temperature region corresponds to the reduction of iron ions incorporated into the perovskite structure.^63–65^ However, it should be noted that iron oxide reduction is a multistage process; thus, some paths can proceed even at higher temperatures.^66,67^ It should also be added that the reduction of iron oxides—FeO, Fe_2_O_3_ and Fe_3_O_4_ occurs within comparable temperature ranges, and the differences in the shape of TPR curves are not substantial. Consequently, it is difficult to definitively identify which iron oxide phases are present in the materials from the TPR profile alone. For the material sintered at 1200 °C, the fraction of iron present as oxides is slightly greater than that incorporated into the SrTiO_3_ structure, as indicated by the larger TPR peak area in the low-temperature region. It should be noted that oxides may often exist as species that strongly interact with the support, in this case with SrTiO_3_. The reduction of such oxides typically occurs at higher temperatures than those required for other oxides crystallized directly on the support surface.^68^ Although the overall shapes of the TPR I and TPR II profiles are similar, noticeable differences appear across both temperature regions. In the low-temperature region, the reduction is largely reversible, as the peak positions in TPR I and TPR II are nearly identical. In contrast, above 600 °C, the peak maximum shifts to lower temperatures during the second reduction. This shift suggests a partial reconstruction of the structure and an easier reduction of iron incorporated into the SrTiO_3_ structure upon subsequent reduction. It should also be noted that the possibility of a reduction of small amount of Fe–Ti–O phases postulated based on the XRD data cannot be excluded. For Fe-doped materials sintered at 1400 °C, a higher proportion of iron is reduced above 600 °C than in materials sintered at 1200 °C, indicating that a higher sintering temperature promotes more effective incorporation of Fe into the SrTiO_3_ structure. It is in agreement with the XRD results, implying the lack of unidentified phases. During the second reduction, the peak maxima associated with the reduction of structure-incorporated iron shift toward lower temperatures, similarly to the behavior observed for the material sintered at 1200 °C. Additionally, the TPR II of the material sintered at 1400 °C shows an unusual distortion of the boundary between the low- and high-temperature reduction regions. This may indicate a partial irreversibility of the reduction of structure-incorporated iron and a tendency for Fe to leave the perovskite structure. This phenomenon is described in the literature as the exsolution process.^69,70^

The TPR profiles recorded for Co-doped SrTiO_3_ are shown in Figure 5c. As in the case of Fe-modified materials, two reduction regions can be distinguished: a low- and a high-temperature region. However, in this system, an atypical increase of H_2_ consumption above 750–800 °C is also observed. A similar effect was previously observed in the Mn-doped sample, but here it occurs regardless of the sintering temperature. For materials sintered at 1200 and 1400 °C, the peaks below approximately 620 °C can be attributed to the reduction of cobalt oxides, since according to the literature, CoO/Co_2_O_3_/Co_3_O_4_ are usually reduced below 600 °C.^71,72^ This reduction is highly reversible, as indicated by the similar shape and intensity of the corresponding peaks in subsequent reductions. Significant differences between the materials sintered at 1200 and 1400 °C are observed in the high-temperature region (620–800 °C), which is associated with the reduction of cobalt incorporated into the perovskite structure, as previously reported in the literature.^39,73^ For the material sintered at 1200 °C, this process is not fully reversible, as the intensity of the reduction peak is noticeably lower in the second reduction. Concurrently, in the low-temperature range, an additional reduction effect appears, suggesting that cobalt was exsolved from the perovskite structure is reoxidized (during the TPOx step) to a cobalt oxide that interacts strongly with the SrTiO_3_-based support, which shifts its reduction to slightly higher temperatures than those of typical cobalt oxides. It should be emphasized that after sintering at 1200 °C, the share of Co incorporated into the perovskite structure is much greater than the share of Co involved in the formation of oxides, as reflected in the substantially higher peak intensities in the high-temperature range. In contrast, the absence of high-temperature reduction peaks for material sintered at 1400 °C suggests that cobalt is no longer present in significant amount within the perovskite structure. The observation is consistent with the possibility of cobalt volatilization during high-temperature treatment, as reported in the literature,^39^ and is supported by the blue color of the alumina crucible used during sintering, suggesting the formation of the Al–Co spinel phase. These findings indicate that 1400 °C is too high temperature for the successful synthesis of Co-doped SrTiO_3_. Importantly, the partial release of cobalt from the perovskite structure leads to structural distortion and the generation of additional point defects, which may explain the significant change in the SrTiO_3_ lattice parameter discussed earlier. Additionally, the gradual increase in the TPR baseline may be attributed to the presence of oxygen vacancies. This effect is clearly noticeable in materials sintered at both 1200 and 1400 °C. It suggests that the concentration of oxygen vacancies in Co-modified SrTiO_3_ is substantial. These defects may arise not only from high-temperature sintering but also from charge compensation associated with Co-modification and strontium deficiency.

The TPR profiles of Ni-doped strontium titanate are presented in Figure 5d. A significant difference between the curves recorded for materials sintered at 1200 and 1400 °C is observed. For the material sintered at 1200 °C, the profiles have a typical two-region shape, with a low-temperature peak (below 600 °C) corresponding to the reduction of NiO, and a high-temperature region (above 600 °C) reflecting the reduction of nickel incorporated into the SrTiO_3_ structure. It corresponds to the previous reports.^42,74^ As observed for Fe- and Co-doped materials, the reduction in the low-temperature region is highly reversible. In contrast, the reduction of nickel incorporated into the perovskite structure is only partially reversible, as indicated by a shift in peak maximum to lower temperatures in the second TPR cycle and the appearance of an additional peak at approximately 420 °C, attributed to the reduction of NiO. The reduction of the oxide at such a low temperature indicates that NiO is very finely distributed. This behavior is consistent with the exsolution of metallic Ni from the perovskite structure during reduction. It leads to the formation of spherical, randomly distributed Ni-species,^45,75^ which are subsequently reoxidized (during the TPOx process) to fine-grained nickel oxide. The substantially larger peak area in the high-temperature region indicates that most of the Ni is incorporated into the perovskite structure rather than involved in the formation of oxide. Surprisingly, the TPR profiles of the materials sintered at 1400 °C do not exhibit distinct reduction peaks, especially at low temperatures (below 500 °C). This suggests the absence of oxide forms (NiO/Ni_2_O_3_) in the material and implies that a larger fraction of Ni was incorporated into the perovskite structure during sintering at 1400 °C, in contrast to lower sintered materials, for which the presence of the nickel oxide phase was unquestionable. Unexpectedly, there are no clear visible reduction peaks in the high-temperature range, suggesting the absence of reducible nickel species in the analyzed system. Unlike cobalt, nickel does not tend to evaporate at such temperatures; thus, this observation is unusual. The gradual increase in the TPR baseline is usually attributed to H_2_ consumption associated with oxygen vacancy formation, as mentioned earlier. However, the appearance of a small peak in TPR II indicates that other factors should also be considered. For high-density materials, gas diffusion is limited; thus, the reduction can be shifted to higher temperatures. The porosity of the Ni-doped SrTiO_3_ sintered at 1400 °C is not significantly different from that observed for Fe or Co-doped materials, but the grain size is larger. Thus, this microstructural effect can be responsible for an increase in the reduction temperature of nickel species in the material sintered at 1400 °C. It should also be remembered that the materials sintered at 1400 °C may contain numerous point defects, particularly oxygen vacancies. Consequently, the formation of defect complexes (e.g., dopant in Ti-site–oxygen-vacancy complexes) can be expected. Thus, the stronger interaction of nickel species within the perovskite structure can result in shifting their reduction to higher temperatures. Therefore, these two factors (i) limited gas diffusion and (ii) the formation of defect complexes may be responsible for the shift of the peaks associated with the reduction of nickel species toward higher temperatures.

Several general conclusions can be drawn from the analysis of the reduction profiles of nonstoichiometric SrTiO_3_ doped with Fe, Co, and Ni: (i) all materials contain Fe/Co/Ni oxides species, despite their absence in the XRD results, (ii) the reduction of Fe, Co and Ni incorporated into the perovskite structure is only partially reversible, (iii) Co and Ni exhibit higher tendency to incorporate into the SrTiO_3_ structure than Fe (for materials sintered at 1200 °C), (iv) sintering of nonstoichiometric SrTiO_3_ doped with Co/Ni at relatively high temperature leads to unusual dopant behavior. It should also emphasize that the TPR profiles of Mn-modified materials differ substantially from those of materials doped with Fe, Co, or Ni. This observation supports the hypothesis that Mn preferentially occupies the Sr-site rather than the Ti-site in nonstoichiometric SrTiO_3_.

To get a deeper insight into the reduction mechanism of the doped material, the samples after the second reduction were subjected to XRD analysis (Figure 6). This postmortem characterization indicates partial degradation of most materials (except for Mn-doped ones) after the reduction–oxidation–reduction cycle, evidenced by the formation of a small amount of TiO_2_ (not exceeding 2.0%). Since no structural changes were observed in Mn-modified materials after the reduction, the minor unidentified Mn–Ti–O phases present in the material sintered at 1400 °C are also assumed to be resistant to the reduction under applied conditions. Additionally, in both Ni-doped materials, approximately 0.3% of metallic nickel was also detected. Also, the post-TPR samples were further examined by SEM coupled with EDS (Figures S4–S7 Supporting Information). SEM microphotographs of fracture surfaces of samples after reduction indicate that only Ni-doped materials degrade during reduction, with local variations in chemical composition and the appearance of areas enriched in Ni and Ti. To better visualize the changes, polished fractures of all materials were prepared and analyzed by SEM-EDS. Representative microphotographs and dopant distribution maps are shown in Figure 6, while the complete data set is provided in the Supporting Information (Figures S4–S7). In general, all materials exhibited some degree of degradation after the reduction–oxidation–reduction cycle; however, these changes were highly localized, and considerable effort was required to find the affected regions.

**Figure 6:**
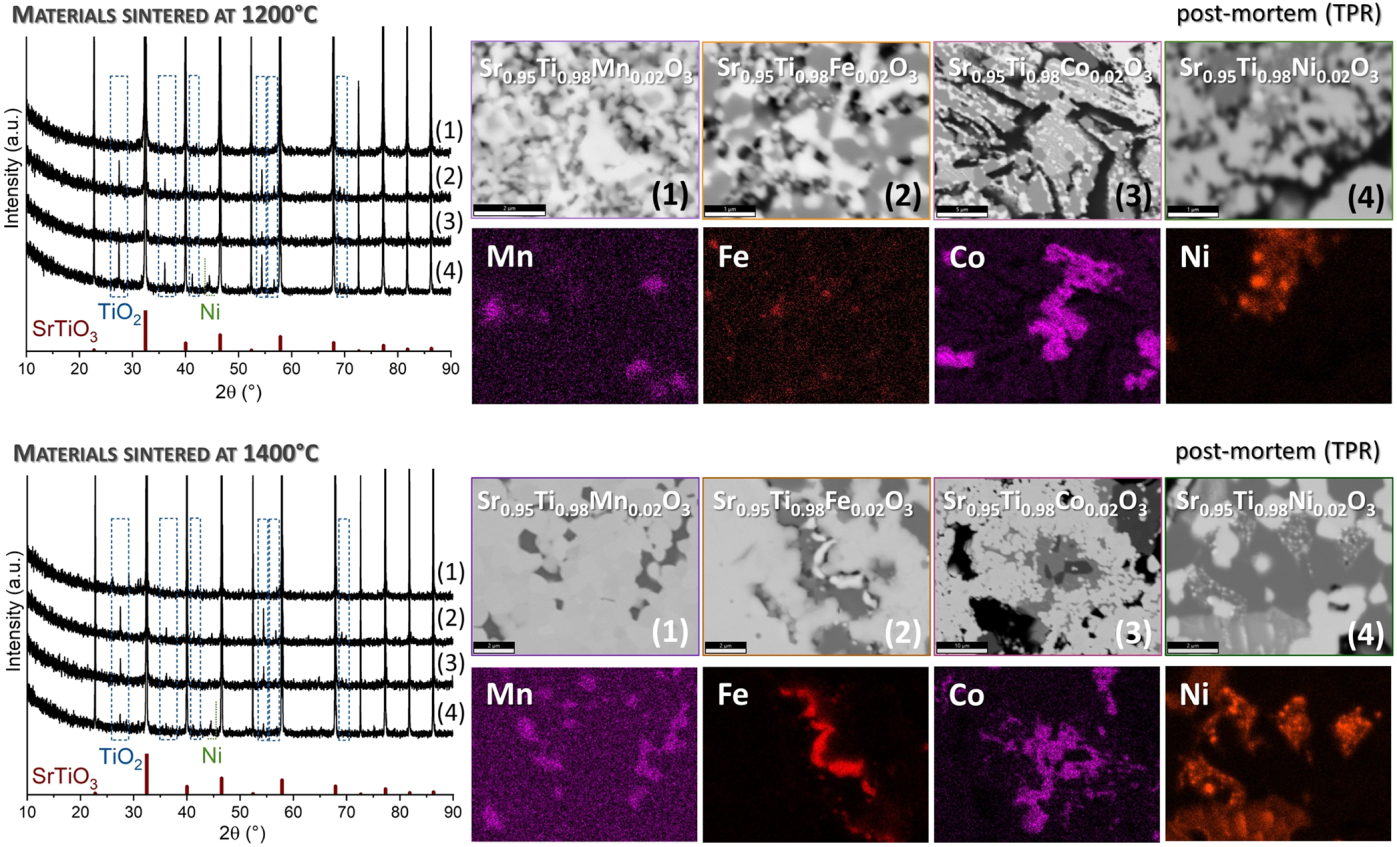
Postmortem analysis of the materials after TPR measurements. The left panel shows XRD patterns of doped SrTiO_3_-based materials; peaks from secondary TiO_2_ are observed within the blue rectangle, while the green rectangle indicates peaks corresponding to metallic Ni. The second panel presents SEM microphotographs and the corresponding EDS distribution maps of Mn, Fe, Co, and Ni, highlighting microstructural and compositional changes caused by the reduction process.

Although the XRD analysis provides no evidence of degradation in the Mn-doped materials, the SEM/EDS results reveal manganese-enriched regions (Figure 6) that also contain a significant amount of titanium (Figure S4). This effect is observed regardless of the sintering temperature. The EDS data suggest that these regions may correspond to manganese-doped titanium oxide or to other unspecified Ti–Mn–O phases, the presence of which was postulated for the material sintered at 1400 °C based on the XRD results. Manganese was also detected within the bright-contrast regions in Figure 6, whose composition corresponds to the perovskite phase. These observations indicate that in the synthesized Mn-doped materials, regardless of sintering temperature, part of the manganese remains incorporated into the strontium titanate structure, even after reduction.

The microstructure and chemical composition of the Fe-doped materials clearly confirm their decomposition upon exposure to hydrogen, irrespective of sintering temperature, as indicated by the XRD results (Figure 6, Figure S5). In most regions, the composition corresponds to Fe-doped strontium titanate; however, separate areas (dark gray contrast) exhibit a composition suggesting the presence of a TiO_2_-based phase, while the bright-contrast regions show substantial Fe content. Elemental distribution maps of Sr, Ti, Fe, and O (Figure S5) clearly indicate that these Fe-rich areas lack Sr, Ti, and O. This supports the hypothesis that these regions represent metallic iron. This finding aligns with previous reports proposing Fe exsolution under reducing conditions.^69,70,76^ Importantly, EDS analysis also shows that not all Fe is removed from the perovskite structure upon reduction, as the perovskite-like phase still contains a small amount of iron.

SEM micrographs of fractures in both cobalt-doped samples after reduction show no evidence of material destruction (Figure 6, Figure S6). Degradation becomes visible only in the polished cross sections. Unlike the iron-doped materials, no metallic (cobalt) inclusions were detected. Although a small amount of cobalt remains incorporated into the perovskite structure (as confirmed by the Co distribution map), a strong tendency for cobalt to form Ti-containing phases (light-gray regions) is evident. At this stage of the study, it is not possible to unambiguously determine whether these regions correspond to cobalt-doped TiO_2_ or to more complex compounds within the Ti–Co–O system. It is also noteworthy that reducing this cobalt-doped perovskite also leads to the formation of TiO_2_ (dark-gray regions). The presence of two various Ti-based phases, including one with significant amount of cobalt, indicates that the reduction pathway of Co-doped strontium titanate is complex.

For nickel-doped samples, material degradation after reduction is the most pronounced, as XRD results already indicated the presence of metallic Ni. SEM microphotographs and EDS analysis clearly confirm the formation of metallic nickel in the form of spherical particles (Figure 6, Figure S7). Interestingly, the Ni distribution maps suggest that nickel is exsolved from the perovskite structure. In addition, besides forming metallic particles, Ni also participates in the formation of an unidentified Ti–Ni–O phase. This behavior was previously observed in cobalt-doped materials, where ternary Ti–Co–O phases were also present. Another similarity between Co- and Ni-doped systems is the formation of a typical TiO_2_ phase (dark-gray regions). Unexpectedly, despite significant differences in the TPR profiles of the nickel-doped samples sintered at 1200 and 1400 °C, the postreduction microstructures are qualitatively similar. This observation supports the interpretations that the gradual increase in the TPR baseline reflects a temperature-shifted reduction of the nickel incorporated into the perovskite structure.

Analysis of postreduction microphotographs clearly indicates that the materials degrade upon exposure to hydrogen. However, the Mn-doped materials are practically unaffected. For the nickel- and iron-doped materials, exsolution of the dopants was clearly observed. Notably, cobalt exsolution was not observed. As expected, one of the phases identified in the postreduction materials was TiO_2_. Interestingly, in the Co- and Mn-doped materials, the TiO_2_ phase was enriched in Co/Mn, suggesting partial solubility of these cations in the TiO_2_ structure. However, it cannot be excluded that, in nonstoichiometric SrTiO_3_, local Me–Ti–O phases (where Me = Mn, Fe, Co, or Ni) may be formed. These phases could also undergo reduction in a hydrogen atmosphere, contributing to the observed postreduction microstructure. It should also be emphasized that the TPR results suggest that cobalt has a high ability to leave the perovskite structure, most likely through evaporation. This behavior is consistent with observations reported in the literature.^77^

It should be noticed that the B–O bond energies (Table S2, Supporting Information) for MnO_2_ and Fe_2_O_3_ indicate greater stability of Mn­(IV)–O and Fe­(III)–O bonds than for Mn­(II)–O and Fe­(II)–O bonds. However, while this is a significant change for manganese, the difference for iron is very small. In the case of Ni_2_O_3_ and Co_3_O_4_ oxides (Co_2_O_3_ was not considered due to a lack of data), the B–O bond energies are less negative compared to those for NiO and CoO oxides, which likely results from a lower tendency for these metals to incorporate into the perovskite structure at higher oxidation states, and higher tendency to stabilize lower oxidation states. It suggests that the structural observations related to the oxidation state of dopants at the B sublattice in the tausonite structure are consistent with calculated B–O bond energies for metals in + II and higher oxidation states (+III, + IV). Also, the TPR results indicate that manganese actually occurs in perovskites in oxidation states higher than + II, iron most probably assumes + III and + II oxidation states in the strontium titanate structure, and for Co and Ni, it is mainly the + II oxidation state.

### Electrical Transport Properties

Figure 7 presents the temperature dependencies of the total electrical conductivity (in dry synthetic air) of doped nonstoichiometric strontium titanate materials sintered at 1200 °C (Figure 7a) and 1400 °C (Figure 7b).

**Figure 7:**
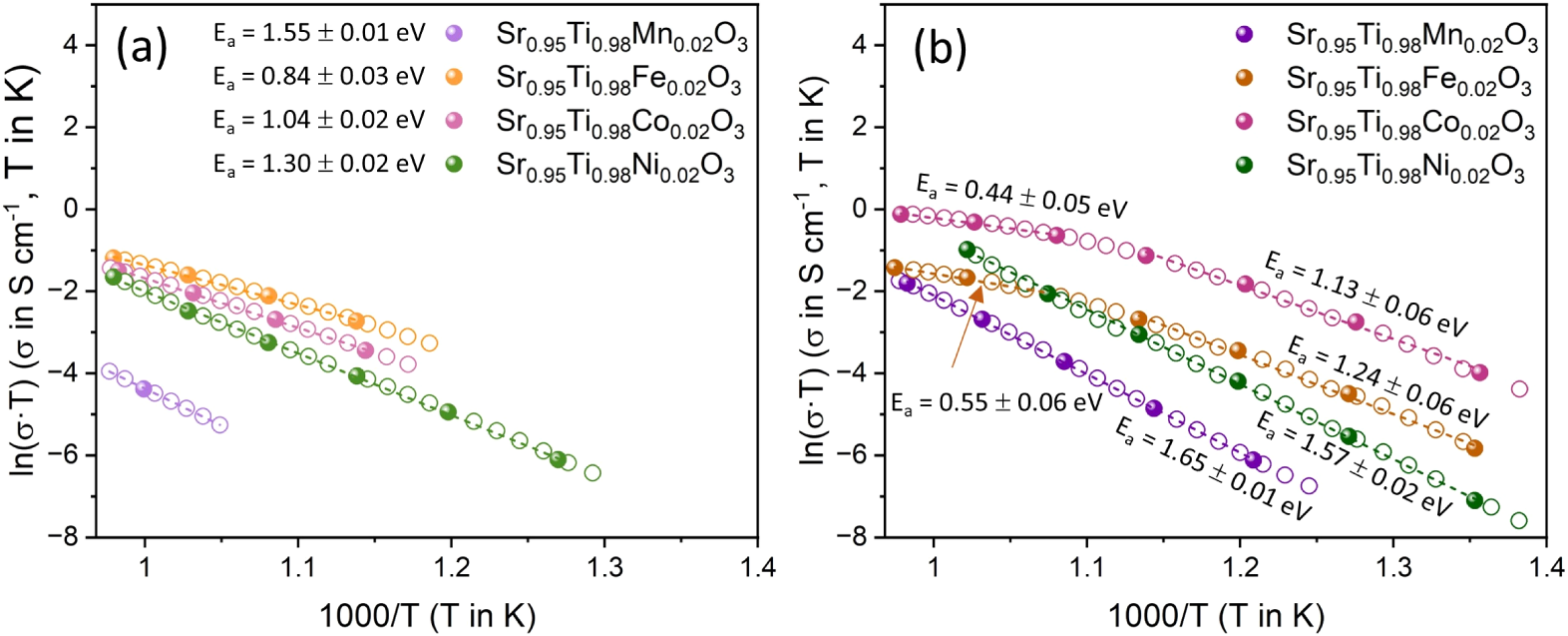
Arrhenius plots of electrical conductivity, ln­(σT), as a function of inverse temperature (1000/T) for doped-SrTiO_3_-based materials sintered at 1200 °C (a) and 1400 °C (b). The measurements were performed in a dry synthetic air atmosphere (purity 99.999%; N_2_ 79%, O_2_ 21%, H_2_O < 3 ppm, CO + CO_2_ < 1 ppm). The fully colored round markers represent data collected after stabilizing the samples at each temperature. These data were used to perform the linear fits (dotted lines) to determine the activation energies (*E_a_*), as indicated. For Mn-modified materials sintered at 1200 °C, all data points shown in the figure were included in the linear fitting procedure.

As shown in Figure 3, the microstructure changes with increasing sintering temperature. Consequently, two main factors must be considered while analyzing the electrical conductivity measurements results: (i) physicochemical properties of the dopant and (ii) the materials’ microstructure. For all materials sintered at 1200 °C, the conductivity values are comparable to or only slightly lower than those of undoped SrTiO_3_ (approximately 10^–3^ S cm^–1^ at 800 °C^78^). This indicates that incorporating 2 mol % of Mn, Fe, Co, or Ni into the perovskite structure does not significantly alter electrical conductivity in oxidizing atmospheres. For all samples, the electrical conductivity increases with temperature, indicating a thermally activated conduction mechanism. It should be noted that in SrTiO_3_ with a deficiency in the Sr sublattice, lattice charge compensation requires the formation of strontium and oxygen vacancies. Acceptor doping (especially with Fe, Co, or Ni) leads to a further increase in the number of defects in the structure. The incorporation of dopant in + II and + III oxidation states into the Ti sublattice, illustrated by eqs 1 and 2, respectively, results in the generation of electron holes:

1 $$NiO\to Ni_{Ti}^{\prime \prime }+2h^{\cdot }+O_{O}^{x}$$

2 $$Fe_{2}O_{3}\to 2Fe_{Ti}^{\prime }+2h^{\cdot }+3O_{O}^{x}$$

However, the charge compensation can also be provided by another mechanism (eq 3), associated with the formation of oxygen vacancies

3 $$O_{O}^{x}+2h^{\cdot }\to V_{O}^{\cdot \cdot }+\frac{1}{2}O_{2\left(g\right)}$$

In the equations above, the Kröger–Vink notation was used, where $O_{O}^{x}$ denotes lattice oxygen, $Ni_{Ti}^{\prime \prime }$ and $Fe_{Ti}^{\prime }$ represent nickel in + II oxidation state and iron in + III oxidation state in the Ti-site, $V_{O}^{\cdot \cdot }$ is an oxygen vacancy and $O_{2\left(g\right)}$ means molecular oxygen. Additionally, at higher temperature the oxygen departure from the perovskite structure can proceed (eq 4):

4 $$O_{O}^{x}\to V_{O}^{\cdot \cdot }+2e^{\prime }+\frac{1}{2}O_{2\left(g\right)}$$

Thus, the formation of oxygen vacancies acts as an electron donor modulating electronic conductivity. Additionally, concerning the potential presence of electron and electron hole, additional process cannot be neglected (eq 5):

5 $$e^{\prime }+h^{\cdot }\to \mathrm{nil}$$

Thus, it should be noted that an acceptor-substituted perovskites can be a mixed ionic-electronic conductor (depending on the atmosphere and temperature), with oxygen ions, electrons, and/or electron holes as predominant charge carriers. However, under oxidation conditions, the dominant charge carriers in the analyzed samples are electron holes and oxygen vacancies, indicating p-type conduction.^78^ The Ni-doped material is expected to exhibit the highest concentration of oxygen vacancies, since Ni usually occupies the Ti-site in the SrTiO_3_ structure in the + II oxidation state, and its oxidation state does not change with temperature. The ionic conductivity of oxygen ions should therefore be the highest for the Ni-doped material. However, within the analyzed temperature range, the electrical conductivity of the Fe-, Co-, or Ni-doped materials sintered at 1200 °C is similar, whereas the Mn-doped material exhibits an order-of-magnitude lower electrical conductivity. Representative electrical conductivity values at 650 °C are summarized in Table 2. Two factors must be considered to explain the lowest electrical conductivity of Mn-doped materials: (i) the oxidation state of Mn incorporated into the strontium titanate structure, and (ii) the microstructure of the Mn-doped material. As postulated from the XRD and TPR results, manganese can be incorporated into both Ti- and Sr-sites, and in both cases acts predominantly as an isovalent dopant. As a result, its incorporation does not strongly affect the perovskite’s defect structure. Furthermore, SEM micrographs (Figure 3) reveal that the Mn-doped material has the smallest grains, resulting in a much higher number of grain boundaries. They can act as a potential barrier to electron–hole transport, significantly reducing charge-carrier mobility and, consequently, the overall electrical conductivity. Activation energies for conductivity are shown in Figure 7 and range from 0.8 to 1.6 eV, depending on composition. Typically, the activation energy for oxygen ion transport via oxygen vacancies for SrTiO_3_ is 0.6–0.8 eV.^79–81^ However, this value can increase due to interactions between defects, including the formation of dopant–oxygen vacancy complexes and/or oxygen vacancy clusters.^82,83^ Thus, the observed values of *E*_*a*_ imply that oxygen vacancies are presented in the analyzed materials. Although the nonstoichiometry in the oxygen sublattice was not directly measured for the presented materials, the presence of oxygen vacancies can be suggested based on the indirect evidence: (i) the values of the activation energy for conductivity, and (ii) the defect chemistry considerations. Also, the relatively high activation energy for the Mn-doped material may be related to the large amount of grain boundaries.

**Table 2:** Values of Total Electrical Conductivity for Doped Nonstoichiometric Strontium Titanate Materials at 650 °C

	Total electrical conductivity at 650 °C (x 10^–4^ S cm^–1^)							
	Mn-doped		Fe-doped		Co-doped		Ni-doped	
	1200 °C	1400 °C	1200 °C	1400 °C	1200 °C	1400 °C	1200 °C	1400 °C
Dry air	0.02^a^	0.3	1.3	1.4	0.8	5.7	0.4	1.4
Dry H_2_	56.1	75.5	7.5	10.3	26.9	465	6.1	23.5

a Estimated value.

For samples sintered at 1400 °C, an increase in electrical conductivity in air is observed, especially for Mn- and Co-doped materials. This effect is most likely associated with changes in the materials’ microstructure, as for these materials, the most significant reduction in porosity is observed (Figure 3). The influence of microstructure on electrical performance is further supported by the behavior of Fe-doped material, which shows only minor changes in both microstructure and electrical conductivity under oxidizing conditions. The activation energies for conductivities determined for materials sintered at 1400 °C are slightly higher than those obtained for materials sintered at 1200 °C. This increase may be attributed to slower gas transport within the denser material and to reduced reduction/oxidation kinetics at the measurement’s conditions. However, for the Co- and Fe-containing samples, a decrease in the apparent activation energy accompanied by a gradual flattening of the temperature dependence of electrical conductivity is observed in the high-temperature region. This behavior is associated with the thermal reduction of the material, whereby increasing temperature leads to a higher concentration of oxygen vacancies (eq 4). These vacancies compensate for the electron charge carriers by consuming electron holes, which ultimately reduces the total electrical conductivity. An additional contribution to this effect may arise from enhanced electron–hole scattering by phonons at elevated temperatures. Notably, this behavior was not observed for the Mn- and Ni-containing samples. Such characteristic flattening of the temperature-dependent electrical conductivity in cobaltite- and ferrite-based perovskites has been previously reported in the literature.^84,85^

Figure 8 shows SEM microphotographs of Sr_0.95_Ti_1–*x*_M_*x*_O_3‑δ_ materials after electrical conductivity measurements performed in a hydrogen atmosphere. Under these conditions, the material is reduced, oxygen is released from the structure, and the concentration of oxygen vacancies is expected to increase. The rate at which the oxygen vacancies are formed strongly depends, among other factors, on the strength of the B–O bond in the perovskite octahedra.^86^ Changes in the oxygen vacancy concentration can significantly affect grain growth and the final microstructure of the materials, as is commonly observed while the perovskite materials are synthesized under a reducing atmosphere. In the present study, SEM microphotographs were obtained on samples synthesized in air and subsequently exposed to an H_2_ atmosphere during electrical measurements. The measurement temperature (800 °C) was much lower than the synthesis temperature (1200 °C/1400 °C), suggesting that large-scale microstructural changes would be less possible. Nevertheless, given that the TPR results reveal microstructural fluctuations caused by reduction, this issue should be considered, especially since the electrical measurements involve heating the sample to 800 °C and subsequently measuring conductivity during cooling. For all materials sintered at 1200 °C, exposure to hydrogen does not lead to significant changes in the microstructure (Figure 8). In contrast, a substantial change is observed for Fe- and Ni-doped materials sintered at 1400 °C. The presence of spherical inclusions rich in Fe and Ni, respectively, suggests that these metals are at least partially released from the perovskite structure as a result of the exsolution process.^45,69^ Interestingly, no analogous behavior was detected in the Co-doped material, despite reports in the literature suggesting that cobalt can also undergo exsolution under reducing atmosphere.^87^

**Figure 8:**
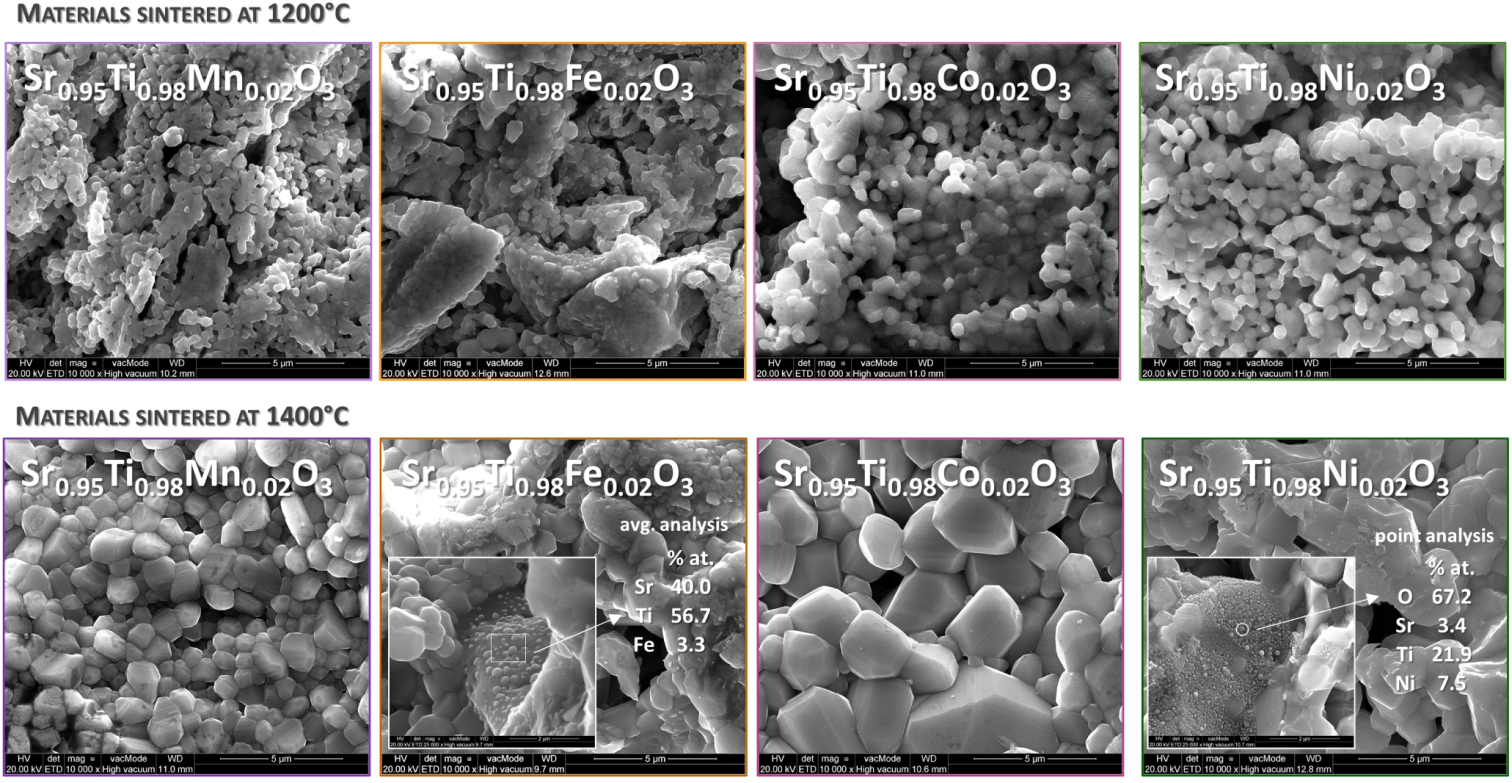
Postmortem microstructural analysis of the doped SrTiO_3_-based material after electrical measurements conducted at an H_2_ atmosphere. The inserts show the Fe- and Ni-enriched inclusions observed in the Fe-and Ni-doped materials, respectively, sintered at 1400 °C.

Figure 9 shows the electrical conductivity of materials sintered at 1200 °C (a) and 1400 °C (b) as a function of temperature measured in hydrogen. For all materials, regardless of sintering temperature, the electrical conductivity values are significantly higher than those measured in air (Figure 7). Under reducing conditions, SrTiO_3_-based materials undergo reduction according to eq 6 (Kröger–Vink notation used):

6 $$H_{2\left(g\right)}+O_{O}^{x}\to H_{2}O_{\left(g\right)}+V_{O}^{\cdot \cdot }+2e^{\prime }$$

**Figure 9:**
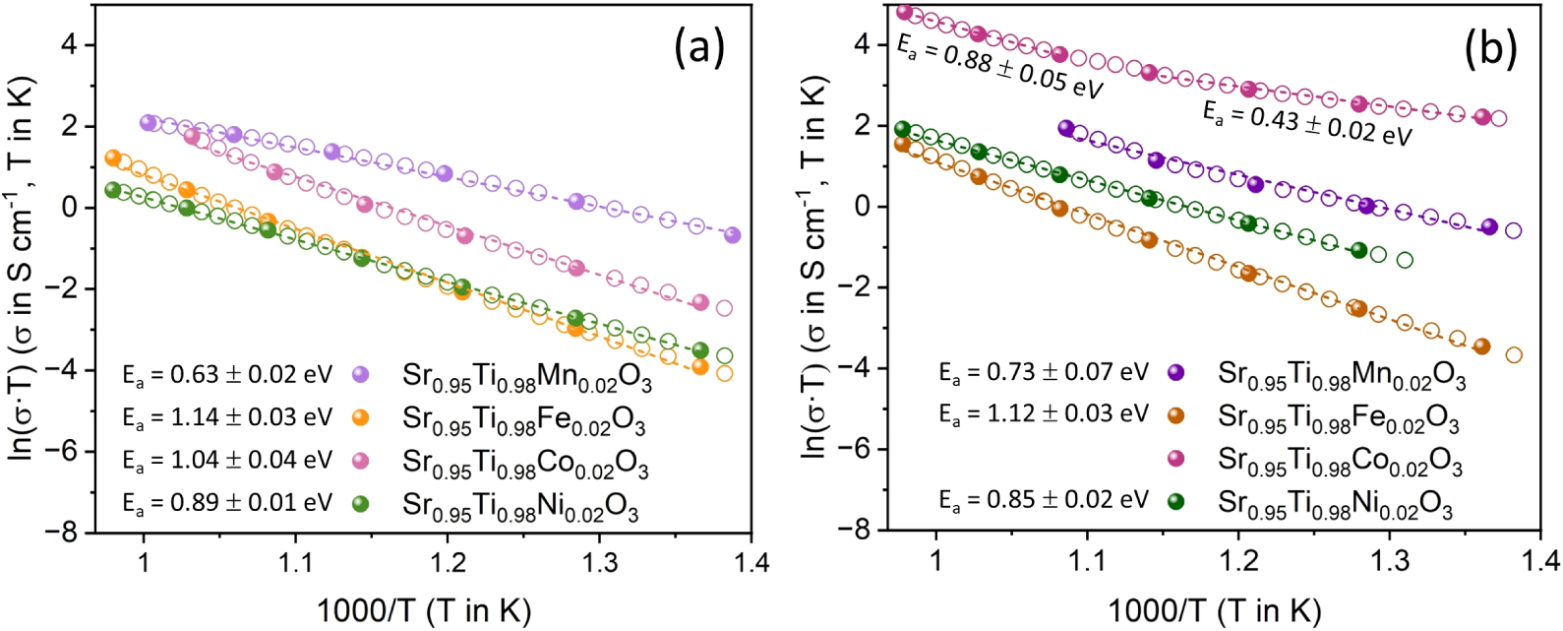
Arrhenius plots of electrical conductivity, ln­(σT), as a function of inverse temperature (1000/T) for doped-SrTiO_3_-based materials sintered at 1200 °C (a) and 1400 °C (b). The measurements were performed in an H_2_ atmosphere. The fully colored round markers represent data collected after stabilizing the samples at each temperature. These data were used to perform the linear fits (dotted lines) to determine the activation energies (*E_a_^*), as indicated.

The oxygen vacancies generated during this process are charge-compensated by electrons (n = $2V_{O}^{\cdot \cdot }$ ), which increases the electron concentration and, as a result, enhances the electrical conductivity. This mechanism is consistent with the higher electrical conductivity values of the materials recorded during measurements in H_2_.

The reduction process is strongly related to the strength of the B–O bond in the BO_6_ octahedra, as weaker bonds favor the migration of oxygen ions in the perovskite structure. The bond energies calculated and presented in Figure 2 are, however, comparable for Co-, Fe-, and Mn-containing compositions. As a result, the differences in B–O bond strength cannot explain the pronounced variations in total electrical conductivity observed in Figure 9. These discrepancies are therefore more likely attributed to other effects rather than intrinsic differences in bond energetics.

For materials sintered at 1200 °C and measured in hydrogen, the Mn-doped material shows the highest conductivity, whereas the Ni- and Fe-doped materials exhibit the lowest. The Mn-doped material exhibits the most significant increase in electrical conductivity when comparing measurements under reducing and oxidizing conditions. It is important to note that Mn can act as an isovalent dopant and, when it does, does not introduce significant defects into the SrTiO_3_ structure and, unlike other dopants, does not generate electron holes. Consequently, no partial compensation of electrons generated under reducing conditions (eq 6) occurs. Therefore, in Mn-doped SrTiO_3_, both electrons and oxygen vacancies contribute to charge transport, leading to an overall increase in electrical conductivity. The activation energies for the conductivity of materials sintered at 1200 °C, shown in Figure 9, were lower than those obtained from measurements performed in air (Figure 7), indicating much higher electron and oxygen-ion mobilities in the structure. The release of oxygen from the structure probably increased the unit cell volume^86,88,89^ and provided more space for oxygen diffusion, resulting in a lower activation energy. The activation energy values for Fe-, Co-, and Ni-doped materials are comparable to those reported in the literature for materials with a high share of oxygen-ion conductivity via oxygen vacancies. In contrast, the activation energy for conductivity for Mn-doped material is much lower, suggesting an increase in the share of electron conductivity. It supports the thesis postulated above about the higher concentration of electrons in this material.

Analysis of samples synthesized at 1400 °C and measured in H_2_ shows that the electrical conductivity of Ni- and Co-doped materials increases compared to that of materials sintered at 1200 °C and measured under the same atmosphere, whereas for Fe- and Mn-doped materials, the conductivity changed only very slightly. It is noteworthy that the largest microstructural changes after reduction in H_2_ were observed in Fe- and Ni-doped materials, where Fe/Ni-enriched particles formed, suggesting an exsolution process. It changes the defect structure in the perovskite phase and is expected to influence the electrical conductivity, since it is connected with the reduction process (eq 7):

$$Ni_{Ti}^{\prime \prime }+2e^{\prime }\to Ni_{\mathrm{metal}}^{0}+{V^{\prime \prime \prime \prime }}_{Ti}$$ 7

The electrons required for the reduction are generated in the reaction given by eq 6; thus, exsolution leads to the creation of titanium vacancies and a decrease in the number of electrons. It can result in a lower share of electron conductivity in the total material conductivity. TPR results indicate that the exsolution process may also occur in Fe- and Ni-doped materials sintered at 1200 °C. Although SEM microphotographs of materials obtained after the electrical measurements do not clearly show exsolved particles, this process, which is difficult to detect in materials with high porosity, cannot be excluded during the reduction in H_2_ before the electrical conductivity measurements. This could explain the lower-than-expected electrical conductivity of Fe- and Ni-doped materials sintered at 1200 °C under H_2_ atmosphere. Also, the activation energy for the conductivity of Fe/Ni-doped materials suggests a significant contribution of conduction via oxygen vacancies, regardless of sintering temperature. It stays in agreement with the discussion provided above concerning the mechanism of exsolution. Surprisingly, the highest electrical conductivity was observed for Co-doped materials sintered at 1400 °C. However, because a fraction of the cobalt could volatilize during high-temperature sintering, a highly defective structure can be postulated for this material, especially since sintering at high temperature favors the reaction given by eq 4. The electrical properties of this material exhibit a strong dependence on the surrounding atmosphere, with a surprisingly low activation energy (0.43 eV) in the low-temperature range in a hydrogen atmosphere. It suggests a small-polaron hopping mechanism for conductivity involving Ti^3+^/Ti^4+^ redox pairs. These observations suggest a partial reduction of titanium ions in the strontium titanate structure. The formation of Ti^3+^ appears to play a key role in maintaining the perovskite lattice and ensuring charge neutrality in the cobalt-depleted system. Also, the removal of the B-site cations can create local strain fields^9^ which can modify the overlap between metal *d*-orbitals and oxygen *p*-orbitals, affecting the mobility of electronic carriers.

Figure 10 summarizes the key properties of Mn, Fe, Co, and Ni (Figure 2) with experimental data (the *E*_*a*_ for conductivity in oxidizing and reducing atmospheres and the temperatures of the maximum TPR peak assigned to the reduction of dopants incorporated into the perovskite structure). For materials sintered at 1200 °C, the qualitative trends between the B–O bond energy and *E*_*a*_ for conductivity in an air atmosphere can be observed, suggesting an influence of the dopant binding strength in the SrTiO_3_ structure on the number of charge carriers generated in the system (both ionic and electronic - eqs 2 and 3). This trend is similar for materials sintered at 1400 °C. However, likely due to cobalt migration, which changes the system’s defect structure, a lower-than-expected *E*_*a*_ for conductivity in air is observed. Also, for materials sintered at 1200 °C, the redox potential appears to be associated with the materials’ reducibility, in particular with the maximum reduction peak; since an increase in the temperature of the maximum reduction peak follows the trend observed for the change in the redox potential of the Me^2+^ + 2e = Me^0^ reaction. Also, the increase in the electronegativity of proper *d*-metals finds reflection in an increase in the material’s reducibility. On the other hand, no clear correlation or qualitative relationship between the reducibility and electrical conductivity of the investigated materials and the Goldschmidt tolerance factor was observed. Although the *t* factor is a fundamental parameter describing the stability of the perovskite structure, the differences in its value among the tested materials are probably too small to affect the materials’ reducibility and electrical properties significantly. It should be emphasized that the analysis of the properties and the search for specific trends in materials sintered at 1400 °C are more complex than for those sintered at 1200 °C, especially due to the Co evaporation effect. As a consequence, no Co-related reduction peak is observed for material sintered at 1400 °C (the corresponding data point is missing in Figure 10). Also, the reduction temperatures for Ni and Mn-doped materials (900 °C) reflect the highest temperature reached during the TPR measurements, rather than the true reduction temperature. Despite these limitations, some association between the B–O bond energy and the activation energy for conductivity in air can be observed, similar to the trend observed for materials sintered at 1200 °C.

**Figure 10:**
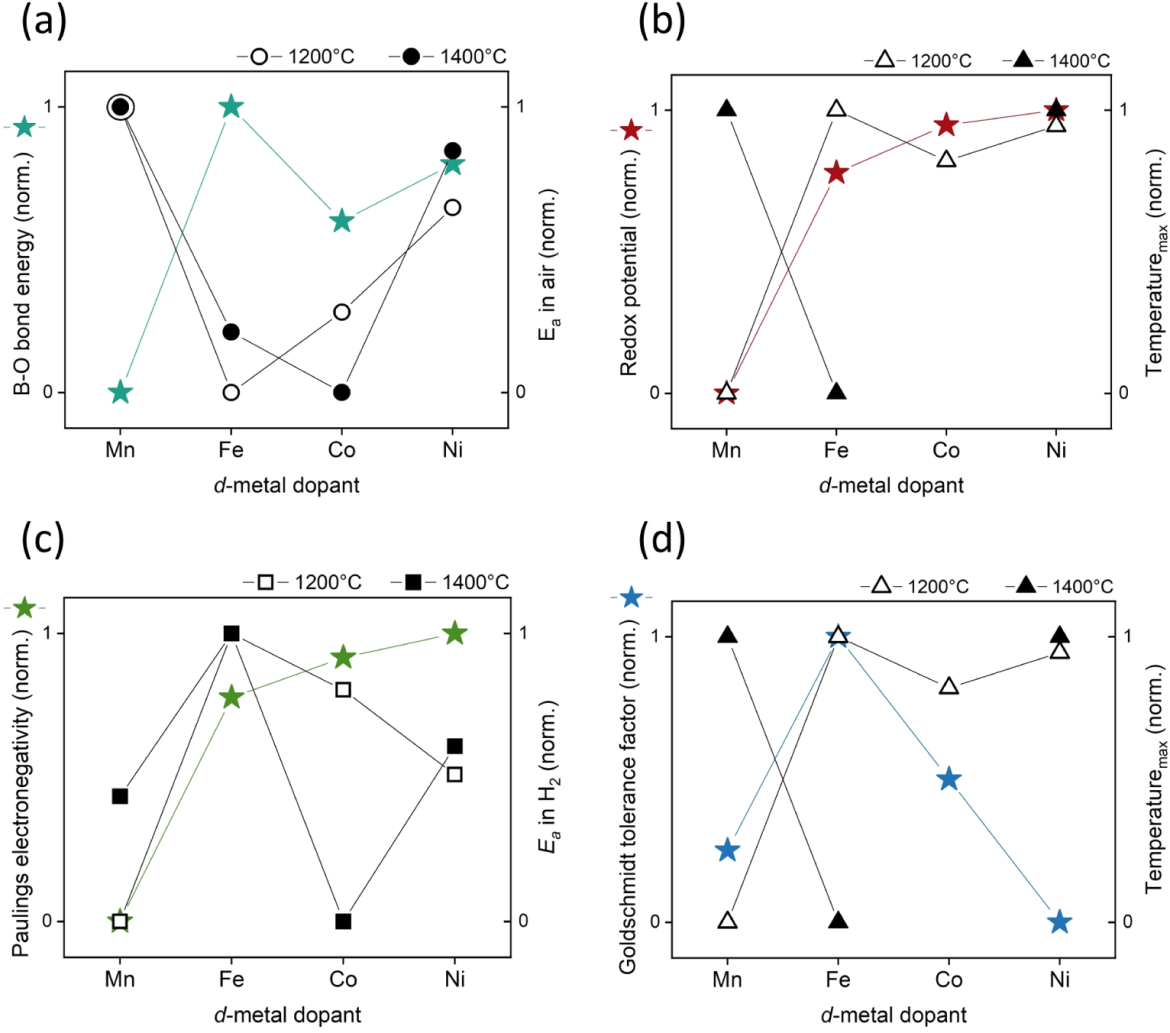
Comprehensive comparison of fundamental dopant properties and experimental data for Mn-, Fe-, Co-, and Ni-doped SrTiO_3_-based materials. Dopant type (Mn, Fe, Co, Ni) is shown on the *x*-axis, while intrinsic properties and experimental data are plotted on the primary and secondary *y*-axes, respectively. The B–O bond energy (a) and Pauling electronegativity (c) are correlated with the activation energies for electrical conductivity in air and H_2_ atmospheres, respectively. The standard reduction potential of the M^2+^/M redox pair (b) and Goldschmidt tolerance (d) factor are correlated with the temperatures of peak maxima in TPR profiles.

Summarizing, the research and analyses suggest that the B–O bond energy and redox potentials play a major role in the transport properties and reducibility of SrTiO_3_-based materials doped with first-row *d*-block metals. However, in the case of materials sintered at a higher temperature (1400 °C), an additional effect associated with Co evaporation (in Co-doped materials) disturbs most of the trends observed in the tested system.

## Conclusions

The investigations and analyses indicate that the transport properties and redox ability of the *d*-metal-doped Sr-deficient SrTiO_3_ can be affected by the B–O bond energy, the dopant’s redox potential, and its electronegativity. However, clear or general relationships between the fundamental chemical properties of the dopants (such as ionic radius, electronegativity, reduction potential, and metal–oxygen bond energy) and the observed changes in structural, microstructural, and functional properties are difficult to establish. The difficulties in establishing these relationships result from both the small amount of introduced dopants and the specific properties (nature) of metals introduced as dopants: (i) Mn has a tendency to migrate between the Sr- and Ti- sublattices, (ii) Fe and Ni can be exsolved from the perovskite structure, (iii) Co evaporates at high temperature, leaving the perovskite structure highly defected. Nevertheless, several conclusions regarding the properties and the performance of the tested materials can be drawn.

- The significant impact of the metal cation dopant on the microstructure of Mn-, Fe-, Co-, and Ni-doped nonstoichiometric SrTiO_3_ was observed. However, the greatest changes in microstructure with temperature were observed for Mn-doped material, which is a consequence of the migration of manganese ions from the Ti sublattice to the Sr sublattice at higher temperatures.
- All tested materials sintered at 1200 °C reversibly undergo reduction processes associated with the reduction of dopant cations incorporated into the perovskite structure. In contrast, for materials sintered at 1400 °C, the dominant reversible process in Mn-, Co-, and Ni-doped materials was the high-temperature reduction of dopants, while in Fe-doped materials, this process involved both the reduction of oxide species and iron ions incorporated into the perovskite structure.
- Postmortem analysis indicates limited stability of materials exposed to an H_2_-rich atmosphere, as evidenced by partial chemical degradation of the materials except the Mn-doped materials, which remain unaffected under these conditions. Additionally, the exsolution of Ni and Fe was observed after exposure of the materials to an H_2_-containing atmosphere.
- The electrical conductivity for all investigated materials in a hydrogen atmosphere is significantly higher than in air, irrespective of the sintering temperature. This behavior is mainly attributed to an increase in the concentration of oxygen vacancies generated in the materials as a result of lattice charge compensation. Still, the effect connected with the reduction of dopants and host Ti^4+^ ions is also observed.

## Supplementary Material



## Notes

Ionic radii on Mn/Fe/Co/Ni ions; calculation details for average ABE bond energy, B–O bond energy, and Goldschmidt tolerance factor; calculated B–O bond energies; scheme of the TPR/TPOx measurements; procedure for electrical property measurements; Rietveld refinement of XRD data; additional postmortem SEM and EDS mapping analysis (PDF)
