# Supplementary material for: Role of First-Row d‑Block Metals in Shaping the Properties of Nonstoichiometric SrTiO3
Source: Inorg Chem. 2026 Jul 21;65(30):17689–705. doi: 10.1021/acs.inorgchem.6c02457 (PMC13439664; doi:10.1021/acs.inorgchem.6c02457)
Supplement: Supplementary file 1 [file ic6c02457_si_001.pdf]

Supporting Information

Role of first-row d-block metals in shaping the properties of non-stoichiometric $SrTiO_3$

Agnieszka Łącz[a], Beata Bochentyn[b], Tadeusz Miruszewski[b], Ewa Drożdż[a]*

[a] Department of Inorganic Chemistry, Faculty of Materials Science and Ceramics, AGH University of Krakow, al. A. Mickiewicza 30, 30-059 Krakow, Poland

[b] Institute of Nanotechnology and Materials Science, Faculty of Applied Physics and Mathematics, Advanced Materials Centre, Gdańsk University of Technology, Gabriela Narutowicza 11/12, 80-233 Gdańsk, Poland

*Corresponding author, e-mail alacz@agh.edu.pl

Table S1 Ionic radii on Mn/Fe/Co/Ni ions as a function of oxidation state for coordination number equal to 6 together with the reference data for titanium [S1].

		oxidation state					
		+II (HS)	+II (LS)	+III (HS)	+III (LS)	+IV	+VII
Mn	ionic radii (Å)	0.97	0. 81	0.785	0.72	0.67	0.60
Fe	ionic radii (Å)	0.92	0.75	0.785	0.69	0.725	
Co	ionic radii (Å)	0.885	0.79	0.75	0.685	0.67	
Ni	ionic radii (Å)	0.83		0.74	0.70	0.62	
**Ti**	**ionic radii (Å)**					**0.745**	
	LS – low spin configuration; HS – high spin configuration						

[S1] Shannon RD, Revised effective ionic radii and systematic studies of interatomic distances in halides and chalcogenides. Acta Crystallogr A 1976;32:751–767. https:// doi. org/ 10. 1107/ S0567 7394760015 51

AVERAGE METAL–OXYGEN BOND ENERGY (ABE)

The expression for the average ABE bond energy is:

$$ABE = \frac{X_A}{12m}\left(\Delta H_{A_mO_n} - m\Delta H_A - \frac{n}{2}\cdot D_{O_2}\right) + \frac{X_B}{6m}\left(\Delta H_{B_mO_n} - m\Delta H_B - \frac{n}{2}\cdot D_{O_2}\right)$$

where X_A, X_B, $\Delta H_{A_mO_n}$ and $\Delta H_{B_mO_n}$ are the molar fraction of A-site cation, B-site cation, the heats of formation of A_mO_n and B_mO_n oxides at 298K, respectively. ΔH_A and ΔH_B are the heats of sublimation of metals A and B at 298 K and D_{O_2} is the oxygen dissociation energy.

The first term of the equation informs about the contribution to ABE of the metal A–oxygen bond energy (the part related to the coordination polyhedron around the A cation), while the second term informs about the contribution made by the metal B–oxygen bond energy (related to the BO_6 octahedra). In the case of introducing an dopants (B') into the B sublattice, the second part of the formula, which takes into account the presence of an admixture, takes the form:

$$BO = z\frac{1}{6m}\left(\Delta H_{B_mO_n} - \Delta H_B - \frac{n}{2}\cdot D_{O_2}\right) + (1-z)\frac{1}{6x}\left(\Delta H_{B'_mO_n} - \Delta H_{B'} - \frac{n}{2}\cdot D_{O_2}\right)$$

Table S2. B–O bond energies in doped-$SrTiO_3$ material calculated for the assumed oxidation states of dopant.

Chemical formula	Oxidation state of d-metal	B–O bond energy [kJ·mol]
MnO	+II	-307.14
MnO_2	+IV	-308.42
FeO	+II	-307.19
Fe_2O_3	+III	-307.50
CoO	+II	-307.17
Co_3O_4	+II and +III	-306.84
NiO	+II	-307.18
Ni_2O_3	+III	-306.99

GOLDSCHMIDT TOLERANCE FACTOR

Goldschmidt tolerance factor can be calculated using the general formula:

$$t = \frac{r_A + r_O}{\sqrt{2} \cdot (r_B + r_O)}$$

where: r_A – ionic radius for A cation (Sr) for coordination number CN=12

r_B - ionic radius for B cation (Ti) for coordination number CN=6

r_O - ionic radius for oxygen anions for coordination number CN=6

In this manuscript the theoretical values of *t* were calculated for $Sr_{0.95}Ti_{0.98}Me_{0.02}O_3$ compound (where Me is Mn, Fe, Co or Ni) assuming that the (r_B+r_O) distance is the linear combination of ionic radii of B cations (Ti and proper dopant), according to the formula:

$r_B + r_O = 0.98r_{Ti} + 0.02r_{Me} + r_O$

Additionally, the 5mol% non-stoichiometry was also included in the calculations since the (r_A+r_O) distance was calculated as follows: $r_A + r_O = 0.95r_{Sr} + r_O$. Also it was assumed that the dopant takes +II oxidation states in the perovskite structure, if HS and LS configuration was possible, the ionic radii of Me^{2+} in LS configuration was taken for calculation.

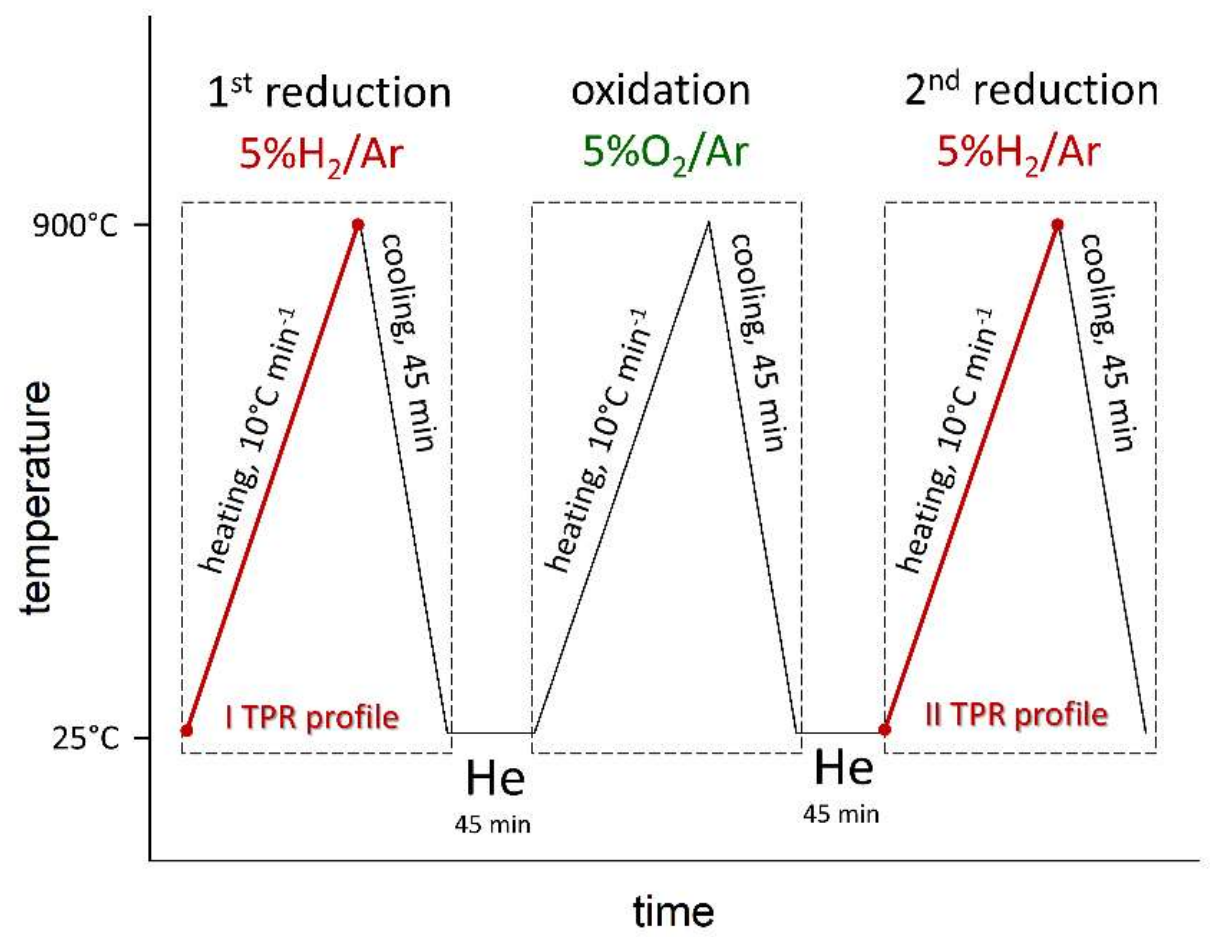

Figure S1. Scheme of the TPR/TPOx measurements.

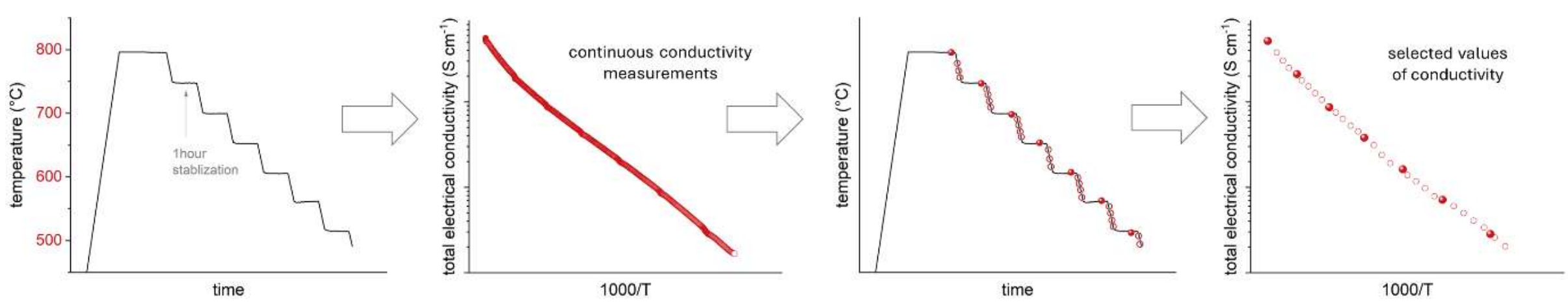

Figure S2. From the measurement to the presentation of the results – the procedure for evaluation of electrical properties of doped-$SrTiO_3$ materials.

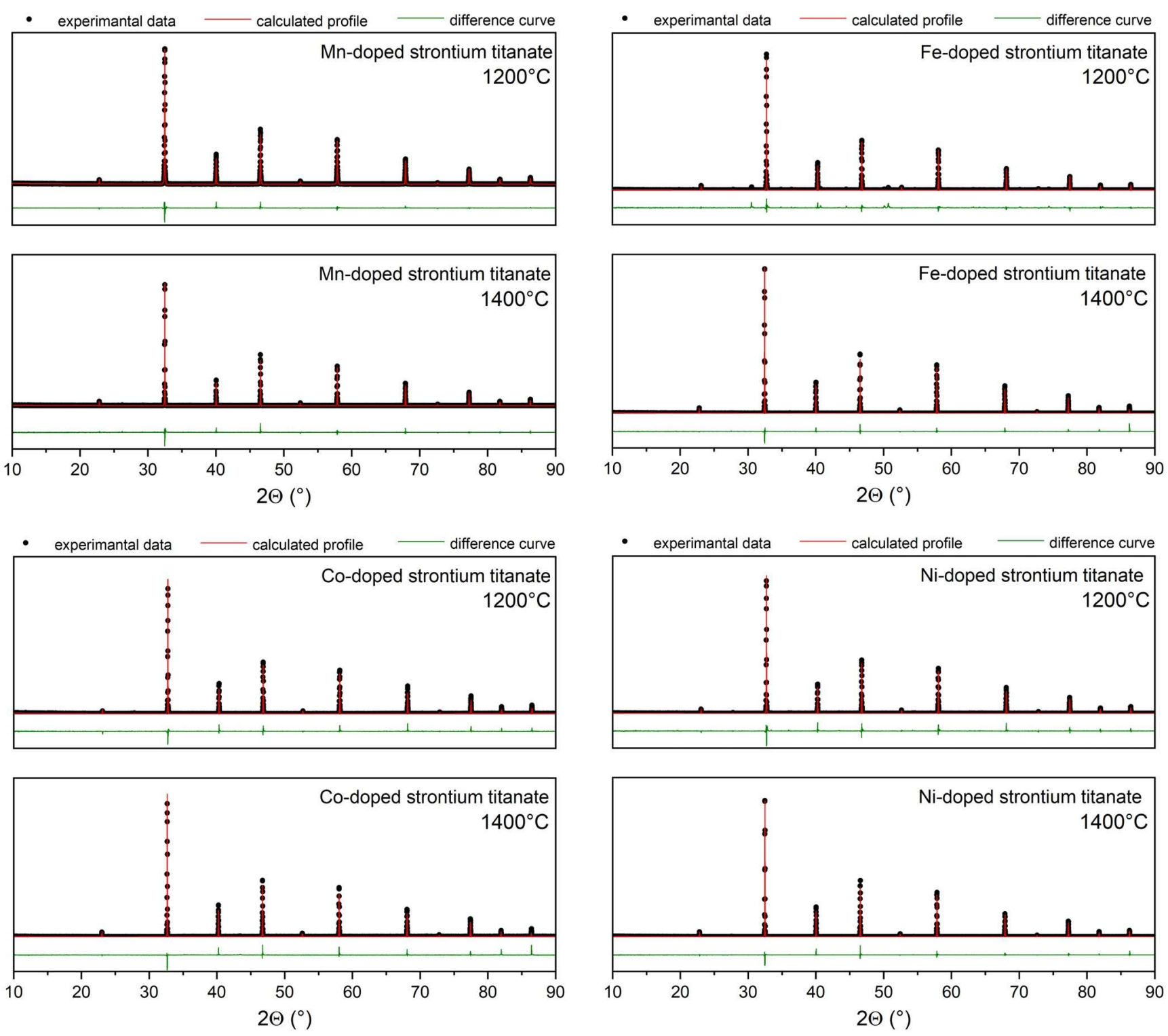

Figure S3. Rietveld refinement of the X-ray diffraction data for all synthesised materials: experimental data (black circles), calculated profile (red solid line), and difference curve (green line, shifted vertically for clarity).

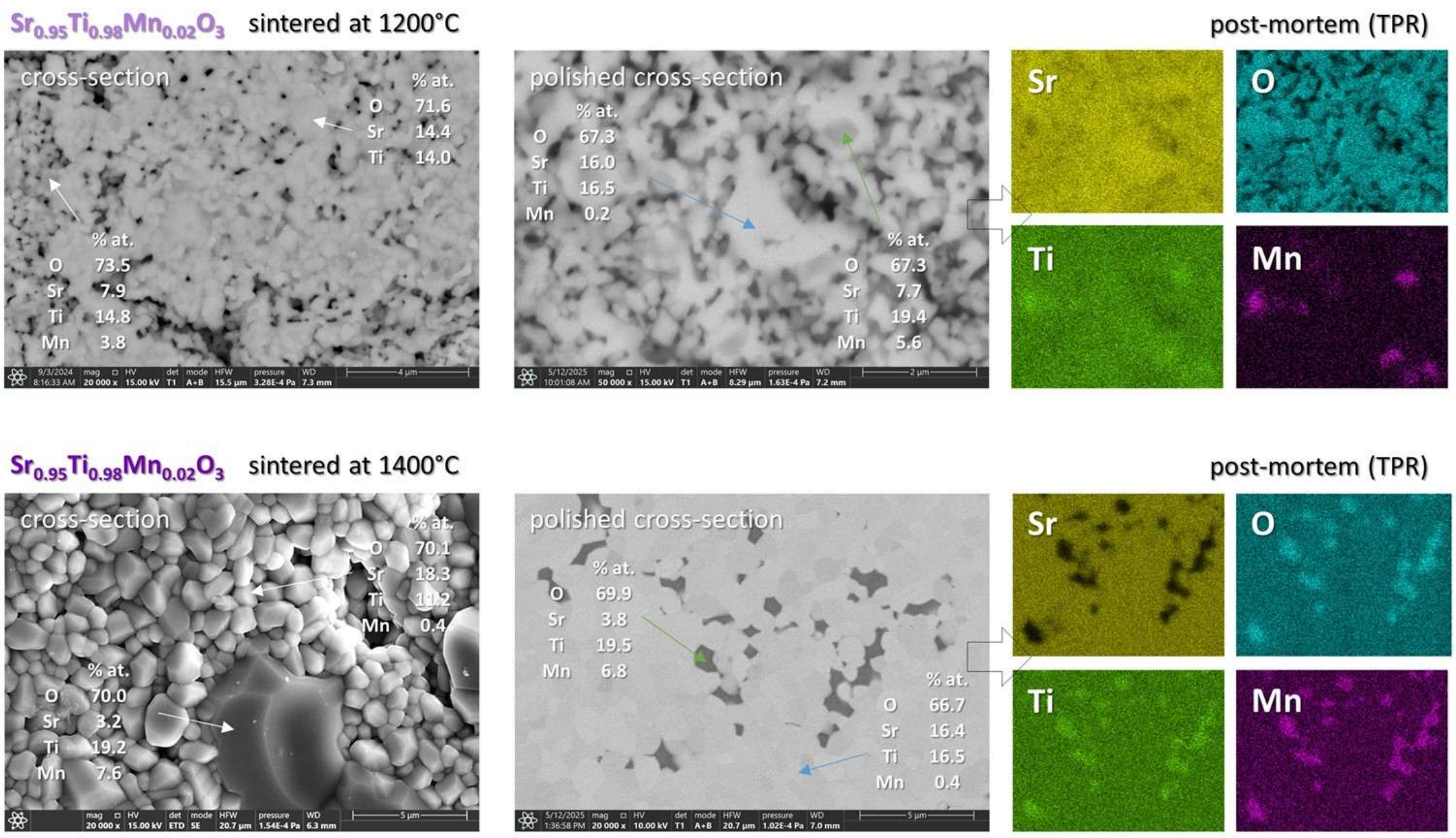

Figure S4. SEM microphotographs and EDS analysis in point and mapping mode for Mn-doped materials after the TPR measurements (post-mortem analysis).

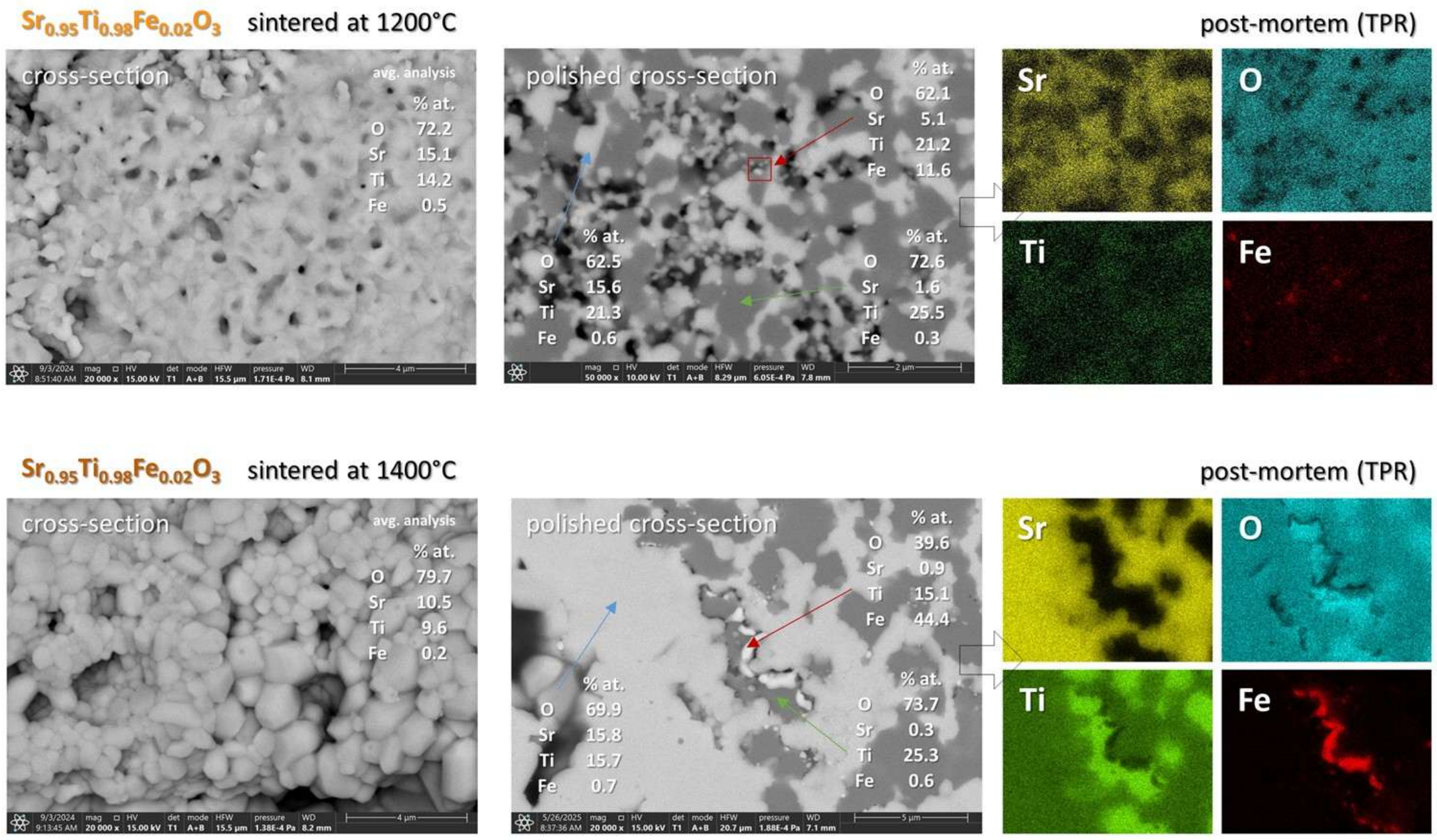

Figure S5. SEM microphotographs and EDS analysis in point and mapping mode for Fe-doped materials after the TPR measurements (post-mortem analysis).

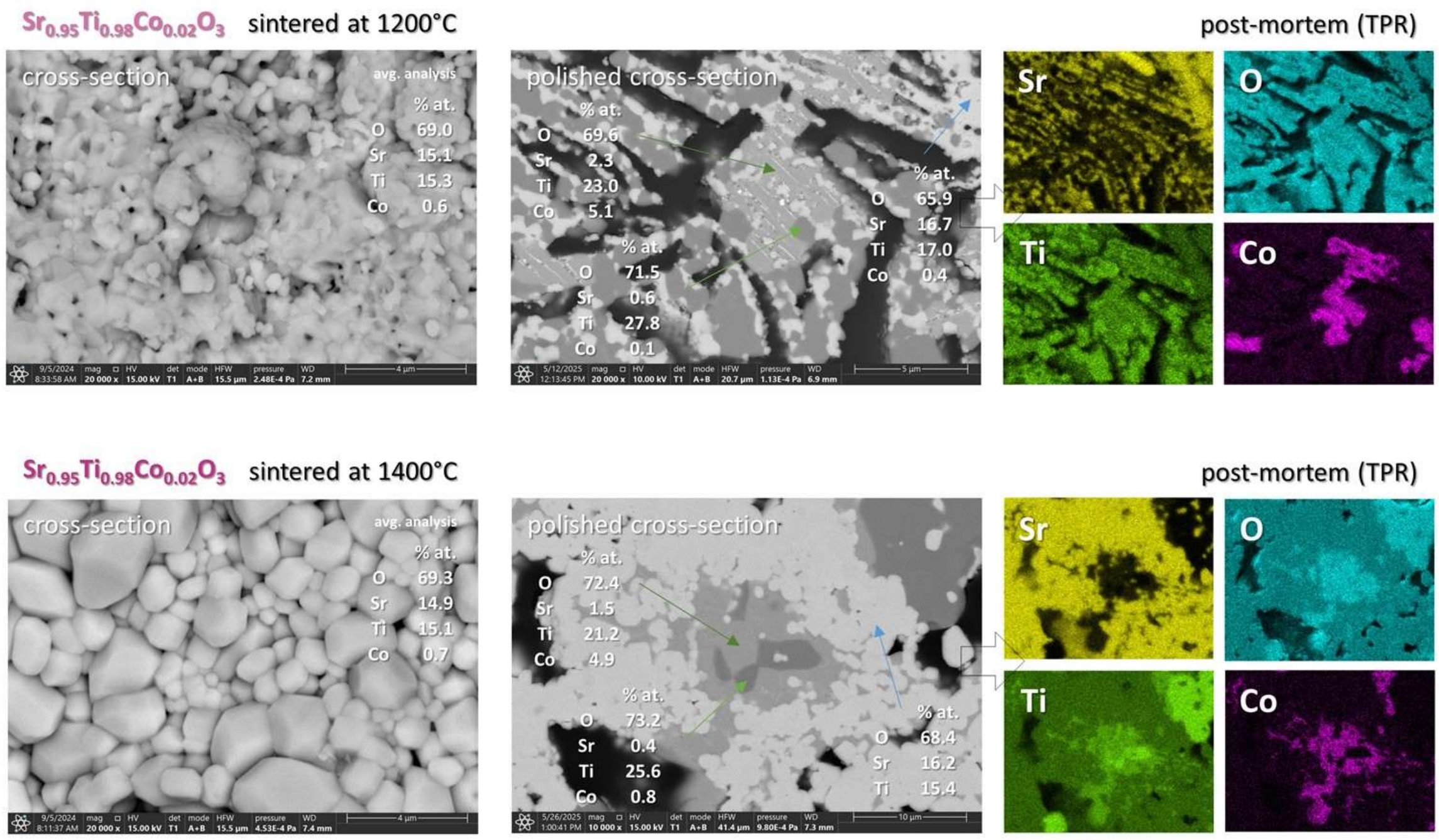

Figure S6. SEM microphotographs and EDS analysis in point and mapping mode for Co-doped materials after the TPR measurements (post-mortem analysis).

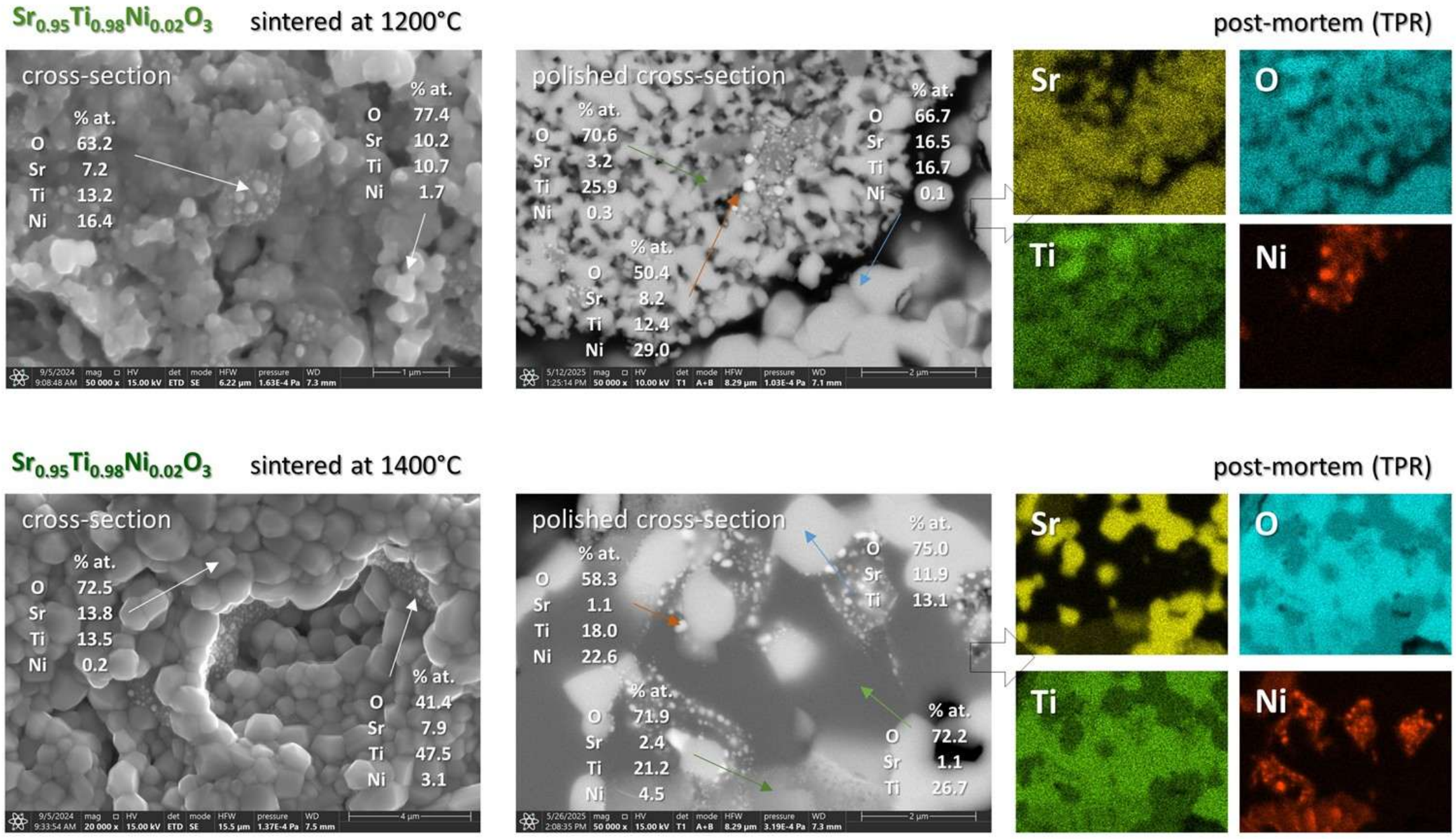

Figure S7. SEM microphotographs and EDS analysis in point and mapping mode for Ni-doped materials after the TPR measurements (post-mortem analysis).
